# The DMD Locus Harbours Multiple Long Non-Coding RNAs Which Orchestrate and Control Transcription of Muscle Dystrophin mRNA Isoforms

**DOI:** 10.1371/journal.pone.0045328

**Published:** 2012-09-21

**Authors:** Matteo Bovolenta, Daniela Erriquez, Emanuele Valli, Simona Brioschi, Chiara Scotton, Marcella Neri, Maria Sofia Falzarano, Samuele Gherardi, Marina Fabris, Paola Rimessi, Francesca Gualandi, Giovanni Perini, Alessandra Ferlini

**Affiliations:** 1 Department of Medical Science, Section of Medical Genetics, University of Ferrara, Ferrara, Italy; 2 Department of Pharmacy and Biotechnology, University of Bologna, Bologna, Italy; 3 Department of Pharmacy and Biotechnology, Health Sciences and Technologies – Interdepartmental Center for Industrial Research (HST-ICIR), University of Bologna, Bologna, Italy; Florida State University, United States of America

## Abstract

The 2.2 Mb long dystrophin (DMD) gene, the largest gene in the human genome, corresponds to roughly 0.1% of the entire human DNA sequence. Mutations in this gene cause Duchenne muscular dystrophy and other milder X-linked, recessive dystrophinopathies. Using a custom-made tiling array, specifically designed for the DMD locus, we identified a variety of novel long non-coding RNAs (lncRNAs), both sense and antisense oriented, whose expression profiles mirror that of DMD gene. Importantly, these transcripts are intronic in origin and specifically localized to the nucleus and are transcribed contextually with dystrophin isoforms or primed by MyoD-induced myogenic differentiation. Furthermore, their forced ectopic expression in both human muscle and neuronal cells causes a specific and negative regulation of endogenous dystrophin full length isoforms and significantly down-regulate the activity of a luciferase reporter construct carrying the minimal promoter regions of the muscle dystrophin isoform. Consistent with this apparently repressive role, we found that, in muscle samples of dystrophinopathic female carriers, lncRNAs expression levels inversely correlate with those of muscle full length DMD isoforms. Overall these findings unveil an unprecedented complexity of the transcriptional pattern of the DMD locus and reveal that DMD lncRNAs may contribute to the orchestration and homeostasis of the muscle dystrophin expression pattern by either selective targeting and down-modulating the dystrophin promoter transcriptional activity.

## Introduction

*DMD* is the largest gene in the human genome; it is 2.2 Mb long, and accounts for approximately 0.1% of the entire human DNA sequence. It consists of 79 exons, 78 introns and of 7 promoters, giving rise to 7 isoforms that are finely regulated in terms of tissue specificity [1]. The three full-length isoforms, denoted B for brain (Dp427b), M for muscle (Dp427m) and P for Purkinje (Dp427p), each contain unique first exons, spliced with a common set of 78 exons, and are highly tissue specific, since the Dp427m isoform is expressed in skeletal and cardiac muscles, the Dp427b isoform is predominantly expressed in the brain (hypothalamus and cortex), but also at low levels in striated muscles, and the Dp427p isoform is mainly expressed in Purkinje cerebellar neurons. The full-length isoforms appear to be developmentally regulated, as the Dp427p isoform is exclusively found in adult tissues, whereas the Dp427m and Dp427b isoforms are also present in human foetal skeletal and cardiac muscle tissues [2]. Four other internal promoters give rise to shorter dystrophin proteins lacking the actin-binding terminus but retaining the cysteine-rich and carboxyl-terminus domains [1].

Mutations in the DMD gene lead to muscle wasting, and two main phenotypes have been defined according to Monaco’s reading-frame theory: the severe Duchenne Muscular Dystrophy, due to out-of-frame mutations, and the milder Becker Muscular Dystrophy, associated with in-frame mutations [3]. A third phenotype, X-linked dilated cardiomyopathy, results from mutations that specifically affect dystrophin transcription in the heart [4]. Heterozygous females for dystrophin mutations are generally asymptomatic, but rarely they can be affected with variable disease severity ranging from mild muscle weakness to a Duchenne-like phenotype [5]. At variance from what historically thought, the symptomatic condition is not related to the X inactivation ratio but seems to be due to other still unraveled expression regulatory mechanisms [6].

Due to the recent advances in drug, molecular and cell therapies [7]–[9] dystrophinopathies are a field of intense interest in terms of both phenotype stratification and dystrophin gene regulation. Particularly, several efforts have been made to address how the dystrophin gene expression is finely modulated, with emphasis on the identification of those factors which are responsible for dystrophin transcription and translation regulation and, therefore, useful for improving/increasing the efficacy of the novel ongoing therapies [10].

Analyses of global transcription profiles have revealed the surprising complexity of the eukaryotic transcriptome, reporting extensive intergenic transcription, antisense transcription, and a considerable number of non-coding transcripts that often overlap or intersperse with multiple coding or non-coding RNAs (ncRNAs) [11].

These ncRNAs have been classified according to their genomic location (coding gene proximity, intronic, intergenic) or transcription orientation (sense, antisense and bi-directional).

ncRNAs have been demonstrated to play a variety of roles, being involved in development [12], control of gene expression (through epigenetic, splicing and transcription mechanisms) and disease modulation [11]–[40]. In addition, long ncRNAs (lncRNAs) have been shown to generate shorter RNAs that can enter the RNAi cascade [41]. However, ncRNAs share common characteristics as they i) are preferentially expressed during embryonic and stem cell differentiation; ii) possess low inter/intra-species sequence conservation (distinguishing them from the highly conserved miRNAs); and iii) are strongly implicated in the mammalian chromatin architecture [42]–[45], with potential effects on the phenotype between and within species [46].

Along with these lines of evidence it would not be surprising whether the complex regulation of dystrophin expression may also result from a combination of factors among which ncRNAs appear to be critical.

To explore this possibility, we interrogated the entire *DMD* gene in the search for non-coding transcripts originating within the dystrophin locus through the design of a novel customised *DMD*-specific gene-expression tiling array. Our results reveal that the *DMD* locus expresses several nuclear long non coding RNAs, characterised by higher-order secondary structure whose function is that of controlling the homeostasis of the dystrophin muscle isoforms expression by targeting the dystrophin promoter regions. These novel data will impact not only on the knowledge about dystrophin gene regulation but also on our understanding on the general mechanisms of action in which regulatory RNA molecules are implicated, not lastly the possibility to use them as biomarkers/modifiers of novel drugs’ response.

## Materials and Methods

### Gene Expression Microarray Design

We tiled the entire *DMD* gene, in both sense and antisense directions, using the web-based Agilent eArray database, Version 4.5 (Agilent Technologies), with 60-mer oligos every 66 bp of repeat-masked genome sequence. We defined probe sets for both orientations, encompassing the *DMD* exons, promoters, introns, predicted miRNA (identified by PromiRII; [47]) and conserved non-coding sequences (CNSs) identified within dystrophin introns using the VISTA programme ([48] http://genome.lbl.gov/vista/index.shtml). Two specific sets of probes were designed to cover, in both directions, the cDNA sequences of a group of control genes (Table S1) identified in the Gene Expression Omnibus (GEO) database [49]) and expressed equally in both normal and dystrophic muscles. Each probe set was opportunely distributed and replicated several times in order to obtain two 4×44k microarrays, referred to as DMD GEx Sense (GEO Platform number: GPL13120) and DMD GEx Antisense (GEO Platform number: GPL13121), respectively, able to detect transcripts in the same and opposite directions as that of *DMD* gene transcription (Table S2).

All the data obtained from these platforms are MIAME compliant and can be accessed from the GEO database [49] by searching GSE27068 in the GEO accession box.

### Sample Processing

Three commercial poly A+ RNAs from normal human brain, heart and skeletal muscle tissues were utilised (Ambion). Skin poly A+ RNA was isolated from total Skin RNA (Stratagene) using the Qiagen Oligotex kit. All RNA samples were checked for purity using a ND-1000 spectrophotometer (NanoDrop Technologies), and for integrity by electrophoresis on a 2100 BioAnalyzer (Agilent Technologies). Sample labelling and hybridisation were performed according to the protocols provided by Agilent (One-Color Microarray-Based Gene Expression Analysis version 5.0.1). The array was analysed using the Agilent scanner and Feature Extraction software (v9.1). Reliability of results was verified via Agilent Quality Controls (Spike-in), consisting of a mixture of 10 *in vitro-*synthesized, polyadenylated transcripts derived from the Adenovirus E1A gene, premixed at concentrations spanning six logs and differing by one-log or half-log increments.

### Data Analysis

**Figure 1:**
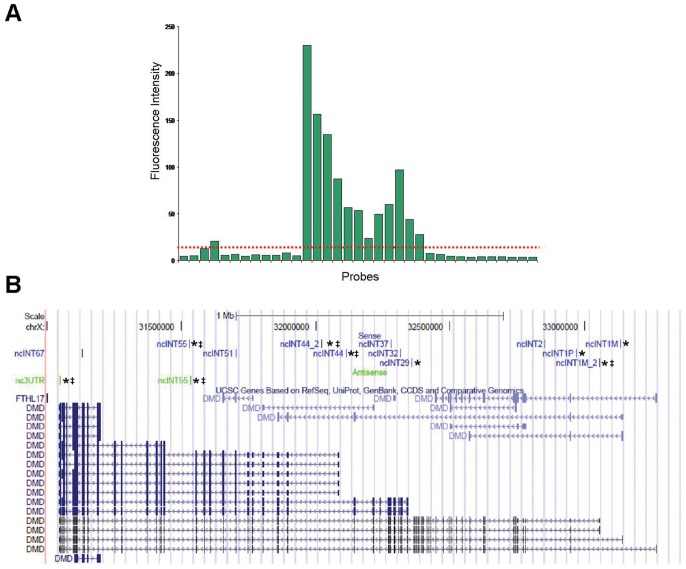
Example of transcripts (ncINT1Ms2) identified by the DMD-GEx array. A) a series of consecutive probes in the genome with fluorescence intensities that rank above the 90th percentile over all probes on the array (indicated with a dashed red line) and mapping within intron 1 M (chrX:33057364-33059742). B) location of the transcripts identified from poly A+ RNA with respect to the *DMD* gene isoforms. Sense transcripts are represented by blue bars, whereas antisense transcripts are indicated by green bars. The transcripts marked with an asterisk and a double cross were characterized by Northern blotting, and RACE PCR.

We considered aggregate transcription consisting of at least three consecutive probes, whose genomic coordinates lay within a 250-nt window, exhibiting fluorescence intensities in the top 90th intensity percentile, as described by Bertone *et al.*, 2004 [50]. In brief, fluorescence intensities for each probe designed within the DMD gene sequence were ranked from the lowest to the highest. Values higher than the fluorescence intensity corresponding to 90% of all ranked probes on the array were considered as positive hybridisation signals. Subsequently, the probes were ordered by their genomic coordinates, and transcripts were identified when at least three consecutive probes in a set of 250 base pairs were above the 90^th^ percentile value (Figure 1 A).

Identified transcripts were named according to their intron of origin, with the prefix ‘nc’ for non-coding and a suffix corresponding to their sense of transcription (‘s’ for sense and ‘as’ for antisense; e.g., ncINT44s is a transcript localized in intron 44 and transcribed in the same direction as the DMD gene).

### Northern Blotting

Sense transcripts originating from the intronic regions harbouring the DMD gene isoform promoters and antisense transcripts were validated by Northern blotting, using a 12-lane human poly A+ RNA filter (Clontech). Riboprobes for ncINT1Ms1, ncINT1Ms2, ncINT1Ps, ncINT29s, ncINT44s1, ncINT44s2, ncINT55s, ncINT55as and nc3UTRas were generated, labelled and hybridized to the RNA filter. Hybridisation and washing were performed according to the manufacturer’s instructions (Clontech), in the presence of herring sperm DNA (10 ug/ul).

### RACE PCR

Rapid Amplification of cDNA Ends (RACE) was performed by using a SMART RACE cDNA Amplification Kit (Clontech), according to adapted manufacturer’s instructions. For both 3′-RACE and 5′-RACE, 1 µg of poly A+ RNA (Ambion) from skeletal muscle (transcripts ncINT44s1, ncINT44s2 and ncINT55s) or heart tissue (transcripts ncINT1Ms2, ncINT55as and nc3UTRas) were reverse-transcribed using PrimeScript reverse transcriptase (TaKaRa). For 3′ RACE, cDNAs obtained from oligo(dT) extension were amplified in three subsequent PCRs in order to increase the degree of reaction specificity. The first amplification was followed by 2 rounds of nested PCRs, performed using Nested Universal Primer (NUP) and a gene-specific primer (GSP2 in the first round and GSP3 for the second). All PCR amplifications were performed using LA Taq (TaKaRa).

For 5′-RACE, first-strand cDNA synthesis was primed using a gene-specific antisense oligonucleotide (GSP-RT), and cDNA was tailed with SMARTII oligonucleotide. 5′-RACE was performed using the same PCR conditions as for 3′RACE.

For three transcripts (ncINT44s1, ncINT55s and ncINT1Ms2), a transcript-walking strategy was adopted, using the sequence that hybridised on the array as an anchor. For both 3′-RACE and 5′-RACE, PCR products were purified and sequenced both directly and after cloning into pCRII vectors using a TA cloning kit (Invitrogen). All primer sequences used for RACE PCR are listed in Table S3.

### Bioinformatics Prediction

The ncRNAs characterized by RACE PCR were tested for secondary structure with mFOLD 3.2 [51], presence of ORF using NCBI ORF Finder [52] and the Coding Potential Calculator [53] and presence of tRNA by TRNAscan-SE 1.21 [54]. To determine whether our transcripts overlapped any previously annotated ESTs or mRNA, we employed the UCSC Genome Browser to inspect our ncRNAs and identify any spliced and unspliced human ESTs and mRNAs.

### lncRNAs Expression and Compartmentalisation Analysis

Total RNA was purified from human RD and SJCRH30 rhabdomyosarcoma cell lines, SH-SY5Y neuroblastoma cell line and both normal and MyoD-induced fibroblasts (transformed with an Ad5-derived, EA1-deleted adenoviral vector carrying the MyoD gene as previously described in [55]) using TRi-REAGENT (Sigma Aldrich) and treating with DNAse I (Ambion). Reverse transcription (RT) was performed using random hexanucleotide primers and Superscript III enzyme (Invitrogen). Real-time PCR was performed in triplicate in a 96-well plate; each 25 µl reaction consisted of 1X Taqman Master Mix (Applied Biosystems), 300 nM forward and reverse primers, 100 nM Taqman probes and 40 ng of cDNA.

To characterise the intracellular compartmentalisation of the ncRNAs, nucleic and cytoplasmic RNA fractions were separated from 1×10^7^ cells (SJCRH30, RD, SH-SY5Y) by glucose gradient centrifugation as previously reported [56], and purified using TRi-REAGENT (Sigma Aldrich). RNA was treated with DNAse I (Ambion), and reverse transcribed using random hexanucleotide primers and Superscript III enzyme (Invitrogen). Real-Time PCR was performed as previously described. The relative quantity of the target sequence (non-coding RNA) is expressed as the relative distribution of each single sequence in the nucleic or cytoplasmic fraction.

Sequences of Taqman probes and amplifying primers are listed in Table S4.

Expression of the different dystrophin isoforms was determined by standard RT-PCR. 250 ng of cDNA derived from total RNA was amplified by PCR using *Taq DNA polymerase recombinant* (Invitrogen) according to the manufacturer’s instructions. Primer sequences are listed in Table S5.

To determine the inclusion of the DMD ncRNAs within the full-length dystrophin transcript, we amplified by RT-PCR the exons adjacent to the introns in which we identified our ncRNAs. The regions amplified were exons 1–3; 28–30; 31–33; 36–38; 43–45; 50–52; 54–56 and 66–68. Primers sequences are available upon request.

### lncRNA Expression Vectors and Transient Transfections in Cultured Human Cells

The expression plasmids pcDNA3.1(+)-ncINT44s, pcDNA3.1(+)-ncINT44s2, pcDNA3.1(+)-ncINT55s and pcDNA3.1(+)-nc3URTas were generated by cloning the PCR product of each ncRNAs into the pcDNA3.1(+) vector (Invitrogen). The template used for PCR amplification was genomic DNA extracted from a healthy human male. Primers listed in Table S6 contain the appropriate restriction enzyme at 5′-end to facilitate cloning.

SJCRH30 Rhabdomyosarcoma and SH-SY-5Y Neuroblastoma cell lines were transiently transfected using Lipofectamine2000 according to manufacturer’s instructions (Invitrogen). Cell cultures were harvested after 48 hours and RNA extracted. Each transfection was repeated three time and each point of RT-PCR was performed in triplicate. Statistical analyses were performed using one-way ANOVA with Dunnett’s test.

### DMD Gene Micro Fluidic Card (FluiDMD v1.1) Analysis

We explored all transcripts originating from the DMD locus, including the lncRNAs identified by our array as well as the whole exome of the dystrophin gene, using a slightly modified design of our FluiDMD card already reported [57]. We replaced the replicated junction systems with specific systems able to detect the following ncRNAs: ncINT1Ms1, ncINT1Ms2, ncINT1Ps, ncINT44s1, ncINT44s2, ncINT55s, ncINT55as and nc3UTRas. The fluidic card protocol was performed as previously described [57]. We used these novel card (FluiDMD-ncRNAs) both in muscle biopsies and cells from dystrophinopathic patients. We analysed seven DMD females’ muscle biopsies. The females were previously selected and known being either symptomatic or asymptomatic carriers (Table 1).

**Table 1:** Classification and mutations of the Female Carriers.

ID	Classification	DMD mutation
**C1**	Symptomatic	t(X;9)(p21.1;p22.1)
**C3**	Symptomatic	Dup exons 5–7 c.265-?_649+?dup (out of frame)
**C4**	Symptomatic	Del exons 8–9 c.650-?_960+?del (out of frame)
**C8**	Asymptomatic	Dup 1P-7 and dup 13–42 chrX:g.(33,068,711_33,068,771)_(32,684,693_32,684,750)dup g.(32,523,766_32,523,826)_(32,228,415_32,228,475)dup
**C12**	Asymptomatic	Del exons 46–51 c.6615-?_7542+?del (out of frame)
**C13**	Asymptomatic	Del exons 49–50 c.7099-?_7309+?del (out of frame)
**C17**	Asymptomatic	c.1615C>T (ex 14) p.R539X

Total RNA was obtained from muscle biopsy of the seven females, carriers of mutations in the DMD gene as previously described [6].

## Results

### DMD-GEx Microarray Data Analysis

Two independent hybridisation experiments were performed with both the DMD-GEx Sense and Antisense microarrays, using poly A+ RNA from human normal brain, heart, skeletal muscle and skin. In both arrays, all genes belonging to the control probe set (Table S1) were expressed with the expected orientation and tissue distribution, to attest the bounty of the hybridization.

Data were normalized in accordance with the Agilent Quality Controls probes (Spike-in) (see Methods), as reported in the literature [58] 90^th^ percentile analysis of fluorescence intensities on both DMD-GEx Sense and Antisense arrays identified as statistically significant a total of 14 poly-adenylated transcripts, (Figure 1 B), which were named according to their intron of origin. As it will be described below none of them contained ORFs encoding for protein longer than 100 amino acids and thereby they were all considered non-coding RNAs (ncRNAs).

Twelve of these ncRNAs, originating from introns 1 M (2 transcripts), 1P, 2, 29, 32, 37, 44 (2 transcripts), 51, 55 and 67, were found to be transcribed in the same orientation as the DMD gene. One ncRNA was found to correspond to the terminal exon and the 3′UTR of the Dp40 isoform [NCBI: NM_004019.2], a known coding dystrophin isoform.

The remaining two ncRNAs, originating from intron 55 (ncINT55as) and from 3′ UTR (nc3UTRas), were found to be transcribed in antisense orientation to the *DMD* gene.

A significant proportion of the identified ncRNAs arose from introns harbouring dystrophin isoform promoters or flanking isoform-specific first exon. In particular, three ncRNAs (ncINT1Ms, ncINT1Ms2 and ncINT1Ps) originated from intron 1 of the Dp427m and Dp427p full-length isoforms, one (ncINT29s) from intron 29 (Dp260), two (ncINT44s and ncINT44s2) from intron 44 (Dp140) and two (ncINT55s and ncINT55as) from intron 55 (Dp116). All identified transcripts were strongly expressed in at least one of the three tissues known to express large amounts of dystrophin. A significant proportion of them were shown to be present in either the skeletal muscle (85.71%) or the heart (71.43%), and nine transcripts were identified in both tissues. Five ncRNAs were found in brain tissue, one of which (ncINT37s) was uniquely expressed in this district (Table 2).

**Table 2:** Genomic location, length and tissue representation of the human transcripts identified.

Poly-A+	Localization	Poly-A+			
ncRNA		Heart	Brain	SkM	Skin
ncINT1Ms	(chrX:33131738-33131978) INT 1M	**−**	**−**	**√**	**−**
ncINT1Ms2	(chrX:33057364-33059742) INT 1M	**√**	**−**	**√**	**−**
ncINT1Ps	(chrX:32969451-32969742) INT 1P	**√**	**√**	**√**	**√**
ncINT2s	(chrX:32853214-32853517) INT 2	**−**	**−**	**√**	**−**
ncINT29s	(chrX:32357565-32357851) INT 29	**√**	**−**	**√**	**−**
ncINT32s	(chrX:32316388-32317177) INT32	**−**	**−**	**√**	**−**
ncINT37s	(chrX:32282002-32282905) INT37	**−**	**√**	**−**	**−**
ncINT44s	(chrX:32112871-32115470) INT 44	**√**	**−**	**√**	**−**
ncINT44s2	(chrX:32019551-32022268) INT 44	**√**	**−**	**√**	**−**
ncINT51s	(chrX:31701831-31702235) Ex/INT 51	**√**	**−**	**√**	**−**
ncINT55as	(chrX:31492064-31533929) INT 55	**√**	**−**	**−**	**−**
ncINT55s	(chrX:31528840-31531268) INT 55	**√**	**√**	**√**	**−**
ncINT67s	(chrX:31129547-31129738) INT 67	**√**	**√**	**√**	**√**
nc3UTRas	(chrX:31047530-31049401) 3′ UTR	**√**	**√**	**√**	**√**
	Total	10	5	12	3
	Percentage	71.4	35.7	85.7	21.4

### Transcript Validation by Northern Blot Analysis

To determine the precise size and relative amount of the ncRNAs, Northern blotting analyses were performed. For this analyses, we selected nine poly A+ sense ncRNAs mapping in proximity to the *DMD* gene promoters or nearby isoform first exons, and two antisense transcripts, mapping at the 3′ end of the DMD locus. ncRNAs whose location within the *DMD* gene suggested a possible regulatory role in the expression of the many *DMD* gene isoforms were preferentially chosen.

**Figure 2:**
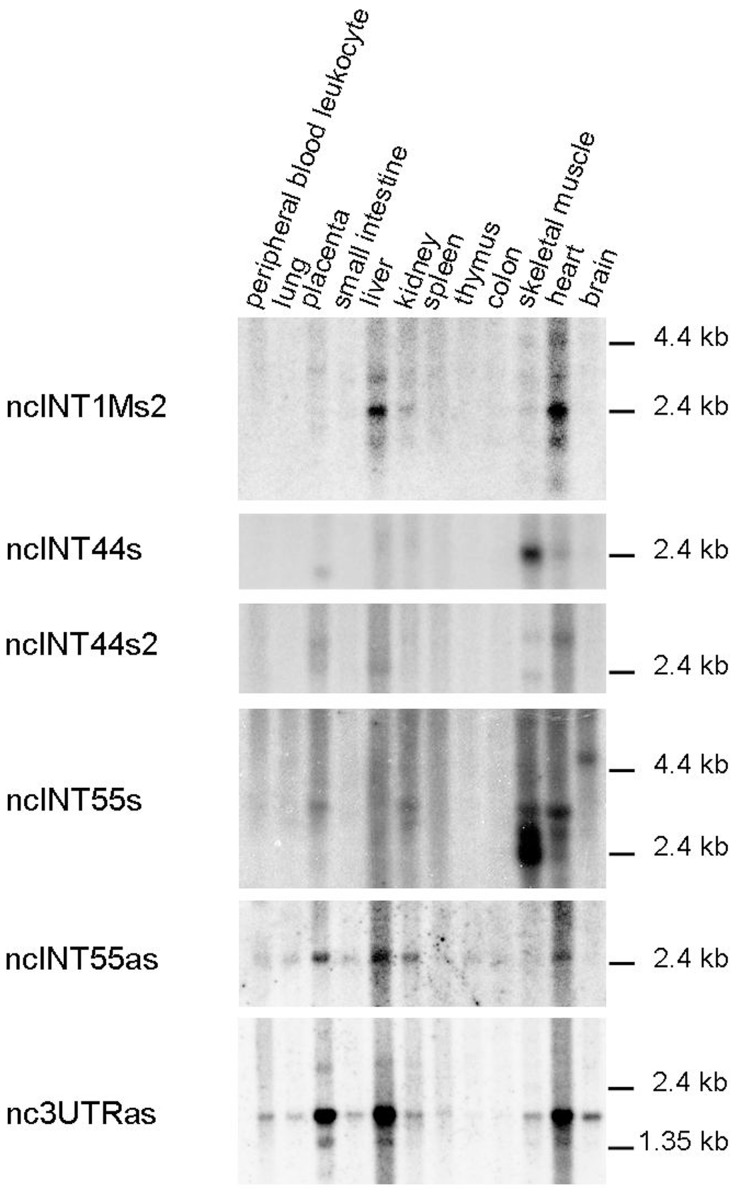
Northern blotting analyses on a 12-lane human poly A+ RNA filter using probes designed on ncRNAs originating near the first exons of *DMD* isoforms and antisense transcripts. Transcript ncINT1Ms2 is located near full-length *DMD* gene isoform Dp427p, whereas ncINT44s and ncINT44s2 surround isoform Dp140. Transcripts ncINT55s and ncINT55as are located upstream the Dp116 isoform. Nc3UTRas overlaps with 3′UTR in antisense direction with respect to the *DMD* gene. All transcripts were expressed in at least one tissue in which DMD isoforms were also expressed, but also in the liver, kidney, spleen and placenta. ncINT1Ms2, ncINT44s2, ncINT55s and nc3UTRas were found to be expressed in multiple isoforms, while one single isoform was detected for ncINT44s and ncINT55as.

Probes identifying transcripts ncINT1Ms1, ncINT1Ps and ncINT29s revealed complex patterns of hybridisation, consisting of multiple bands or a smeared signal, in several tissues (See Figure S1) indicating the possible presence of repetitive elements within the full-length transcripts. Due to the difficulty of defining a precise band on Northern blotting, these ncRNAs were no longer studied. For the remaining four sense and the two antisense transcripts, Northern blotting revealed several distinct transcripts, ranging from 1.4 to 4 kb in length (Figure 2). All ncRNAs were present in at least one of the three tissues in which dystrophin is normally expressed (skeletal muscle (SkM), heart and brain), with a distribution and amount reflecting that observed in the array (ncINT1Ms2, ncINT44s, ncINT44s2 and ncINT55as: SkM and heart; ncINT55s and nc3UTRas: SkM, heart and brain). The ncINT1Ms2, ncINT44s2, ncINT55as and nc3UTRas were highly represented in the heart tissue, whereas the ncINT44s and ncINT55s were found to be more intensively transcribed in skeletal muscle.

Furthermore, most transcripts were also detected in the liver, placenta and/or kidney (Figure 2).

Different tissue-specific isoforms were detected in four transcripts: ncINT1Ms2 presented at least four isoforms, ranging from about 1.8 to 5 kb in size. A 2.4 kb form is prevalent in the heart and liver, whereas it is poorly represented in the skeletal muscle and kidney; two ncINT44s2 isoforms were found in the skeletal muscle tissue, while only the larger one (about 2.7 kb) was detectable in the heart; ncINT55s was detectable in the heart and SkM in at least three different isoforms (from 2.4 to 3 kb), showing a specular pattern of expression in the two tissues. The larger of these isoforms was also found to be present in the kidney and placenta. An additional isoform of about 5 kb is present exclusively in the brain. Nc3UTRas is expressed in the placenta, liver and heart in at least two isoforms (1.5 kb and 1.8 kb). The larger form is widely represented in the other tissues analysed, with the exception of the thymus and colon (Figure 2).

Single forms of ncINT44s and ncINT55as were detected by Northern blotting. NcINT44s is roughly 2.4 kb in size and is expressed almost exclusively in the heart and skeletal muscle, being particularly abundant in the latter, with the occurrence of a shorter isoform being noted in the placenta. In contrast, the ncINT55as transcript is about 2.4 kb in length and displayed widespread distribution, being present in all analysed tissues except the brain and muscle.

### Full-length Transcript Characterisation by RACE PCR

To precisely define the size of the six DMD ncRNAs validated by Northern blotting, 3′ and 5′-RACE analyses were performed on poly A+ RNA.

#### ncINT1Ms2

**Figure 3:**
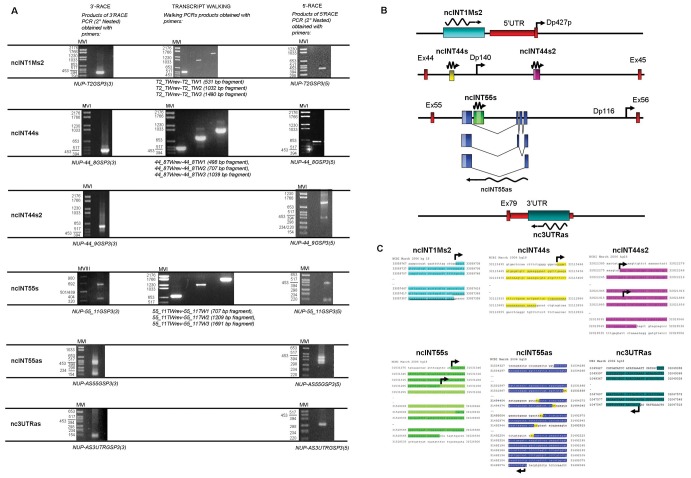
Full-length transcript characterisation by RACE PCR. A) For transcript ncINT1Ms2, a single product of 370 bp was obtained with 3′-RACE. After transcript walking, 5′-RACE was performed and identified a 396-bp PCR product. ncINT44s 3′-RACE generated a single product of 423 bp. After transcript walking, PCR, 5′-end was identified by 5′-RACE, and gave rise to a 599-bp PCR product. For transcript ncINT44s2, a single product of 552 bp was obtained by performing 3′-RACE, whereas 5′-RACE originated two distinct PCR products of 328 bp and 689 bp, corresponding to different transcriptional start sites. 3′-RACE of transcript ncINT55s revealed two products, of 150 bp and 442 bp, corresponding to different 3′ ends of the transcript whereas 5′-RACE gave rise to two distinct PCR products of 673 bp and 352 bp. NcINT55as analysis by 3′-RACE identified two PCR products of 99 and 431 bp, corresponding to different 3′ ends. 5′-RACE identified three PCR products of 307, 343 and 398 bp. Both 3′ and 5′-RACE PCR products for transcripts nc3UTRas showed unique bands of 99 bp and 286 bp, respectively. Relevant pairs of primers used are listed below each image. B) Schematic representations of the RACE PCR results for transcripts ncINT1Ms2, ncINT44s, ncINT44s2, ncINT55s, ncINT55as and nc3UTRas. Transcript orientation is indicated by the zigzag arrows. Their position with respect to adjacent dystrophin exons and isoform promoters is shown. *DMD* gene exons are shown as vertical red boxes, and 5′ and 3′ UTRs as horizontal red boxes. Lighter colours within the transcripts ncINT44s2, ncINT55s and ncINT55as boxes represent alternative starting or polyadenylation sites. The three alternative spliced isoforms of transcript ncINT55as are represented. C) Sequences of the identified transcripts are shown with reference to human genome build 18 (hg18, March 2006). Donor and acceptor splice sites are highlighted in yellow for transcript ncINT55as. Polyadenylation sites are underlined in the sequences. Curved arrows show the starting site for transcription of non-coding transcripts.

The products obtained from 3′-RACE, transcript walking and 5′-RACE were sequenced and combined, generating a full-length sequence of 2379 bp. The ncRNA was sense-transcribed with dystrophin mRNA and was found to be colinear with the dystrophin intron 1 sequence (Figure 3 A, B and C). The 3′ end of the ncINT1Ms2 transcript is located 897 bp upstream of the 5′UTR Dp427p full-length dystrophin isoform.

#### ncINT44s and ncINT44s2

For ncINT44s, 3′ and 5′-RACE both yielded a full-length 2.6-kb transcript. Nucleotide sequence analysis revealed that this RNA was entirely transcribed from intron 44, and did not undergo splicing (Figure 3 B and C). For ncINT44s2**,** the product obtained from 3′-RACE was combined with the two products identified by 5′-RACE, which defined two alternative transcriptional start sites; 2452-bp and 2718-bp sequences were obtained, corresponding to the two full-length isoforms. Sequencing analysis revealed that these RNAs were entirely transcribed from intron 44, without undergoing splicing events (Figure 3 B and C). The two ncRNAs identified within intron 44 are located 29 kb upstream (ncINT44s) and 61 kb downstream (ncINT44s2) of the Dp140 dystrophin isoform promoter.

#### ncINT55s and ncINT55as

For ncINT55s, 3′-RACE identified two different 3′ ends. After transcript walking, 5′-RACE gave rise to two distinct PCR products corresponding to different transcriptional start sites (Figure 3 A). Nucleotide sequence analysis revealed that these transcripts originated entirely from intron 55, without splicing events, (Figure 3 A, B and C). Combination of these start and polyadenylation sites potentially generates at least four transcripts of 2805, 2720, 2513 and 2428 bp, which is consistent with the Northern blotting data. The ncINT55as is transcribed in an antisense direction with respect to transcription of the dystrophin gene. 3′-RACE identified two different 3′ ends, and 5′-RACE detected three isoforms with a common 5′ end (Figure 3 A). Interestingly, these transcripts are alternatively spliced, generating RNAs of different sizes. A shorter isoform carrying only the common 3′and 5′ ends was also identified (Figure 3 B and C). The predicted molecular weight of the three different isoforms ranges from 2642 bp to 2732 bp, being consistent with a single product detectable by Northern Blotting analysis. Notably, ncINT55s residues within that DNA region encode for the largest intron of the mature ncINT55as RNA isoforms. All of these different transcripts identified within intron 55 are located roughly 55 kb upstream of the Dp116 promoter.

#### nc3UTRas

Sequence analysis of the 3′ and 5′-RACE products gave a full-length 1872-bp transcript transcribed in an antisense direction from the 3′UTR region of the DMD gene. Since splicing events were not observed (Figure 3 A, B and C), this transcript overlaps (in an antisense orientation) roughly 2 kb of the 3′UTR region of all full-length and 3′ dystrophin isoforms.

### Bioinformatics Analysis of Coding Potential and Secondary Structure

**Figure 4:**
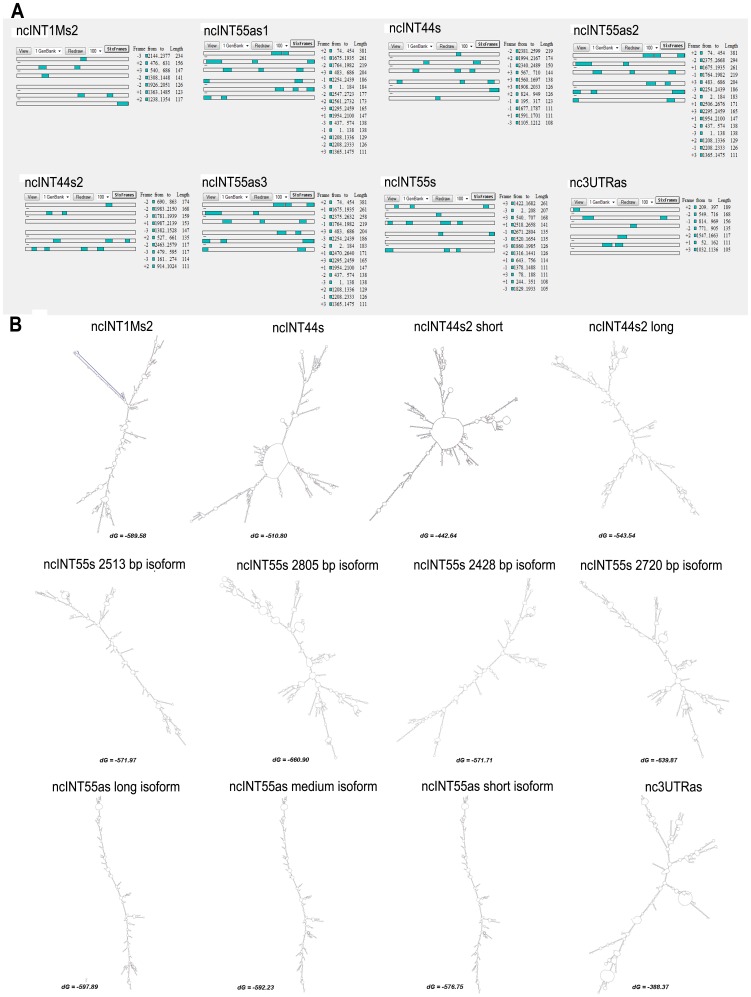
Coding potential and secondary structure bioinformatics analysis. A) ORF prediction. The on-line tool *ORF Finder* was used to detect open reading frames in our lncRNAs. ORFs identified by the programme are shown in green. Each grey line represents the reading frame, divided into sense (first three lines) and antisense (fourth to sixth lines) strands. The length, reading frame, nucleotide start and end position of each ORF are listed on the right of each transcript; B) Mfold analysis of the secondary structure of the lncRNAs characterised: Mfold software showed that all six lncRNAs were able fold into secondary structures with negative ΔG below −50 kcals, indicating the great stability of these structures. Interestingly, ncINT1Ms2 transcript folds in a stem structure (blue line), which accords with the highly conserved sequence shown in Figure 4.

To confirm that the six ncRNAs did not possess the potential to encode for polypeptides, ORF-Finder software was employed to predict putative ORFs with a minimal length of 100 amino acids. As shown in Figure 4 A, none of the three sense-frames contained any ORF fitting those criteria.

The same results were obtained using the CPC tool, which identified a weak coding potential (one ORF of 51 amino acids) for the ncINT1Ms2 (Figure S2). CPC also analysed homology with UTR regions, and found 37 and 35 hits for ncINT1Ms2 and ncINT55as, respectively (File S1).

Using Mfold software, we also determined whether these transcripts could fold into higher secondary structures typical of non-coding RNAs. Interestingly all six ncRNAs can fold into secondary structures with a ΔG below −50 kcals (Figure 4 B), thereby suggesting that these structure may be extremely stable and likely to serve as domains for interaction with other cellular components.

Conservation analysis performed using the UCSC Genome Browser [59] showed that all identified transcripts are poorly conserved, with the exception of nc3UTRas, which is transcribed from the 3′UTR of the DMD gene, and transcript ncINT1Ms2, which presents a highly conserved sequence of roughly 130 bp (Figure S3).

The UCSC Genome Browser was also employed to check for the presence of any known ESTs or mRNAs overlapping DMD ncRNAs. For some of the ncRNAs identified, predominantly small tags intersecting their sequences were detected: ncINT1Ms was found to overlap with part of a wider mRNA found in the uterus [60]; ncINT1Ms2 contained two smaller ESTs found by Robertson et al. [61] in 16-22-week foetal cochleas; ncINT1P overlapped several ESTs previously described [62]–[66] and corresponds to the TBCAP1 pseudogene (ENST00000436520); ncINT32s was found to contain a 9 bp *Gallus Gallus* DMD exon; and ncINT44s and ncINT55s, respectively, overlapped two smaller ESTs identified in neuroblastoma tissues [67].

No tRNAs were detected within the sequences of the ncRNAs identified (data not shown).

### ncRNA Expression Profiles Mirror those of Full-length Muscle and Brain Dystrophin Isoforms

**Figure 5:**
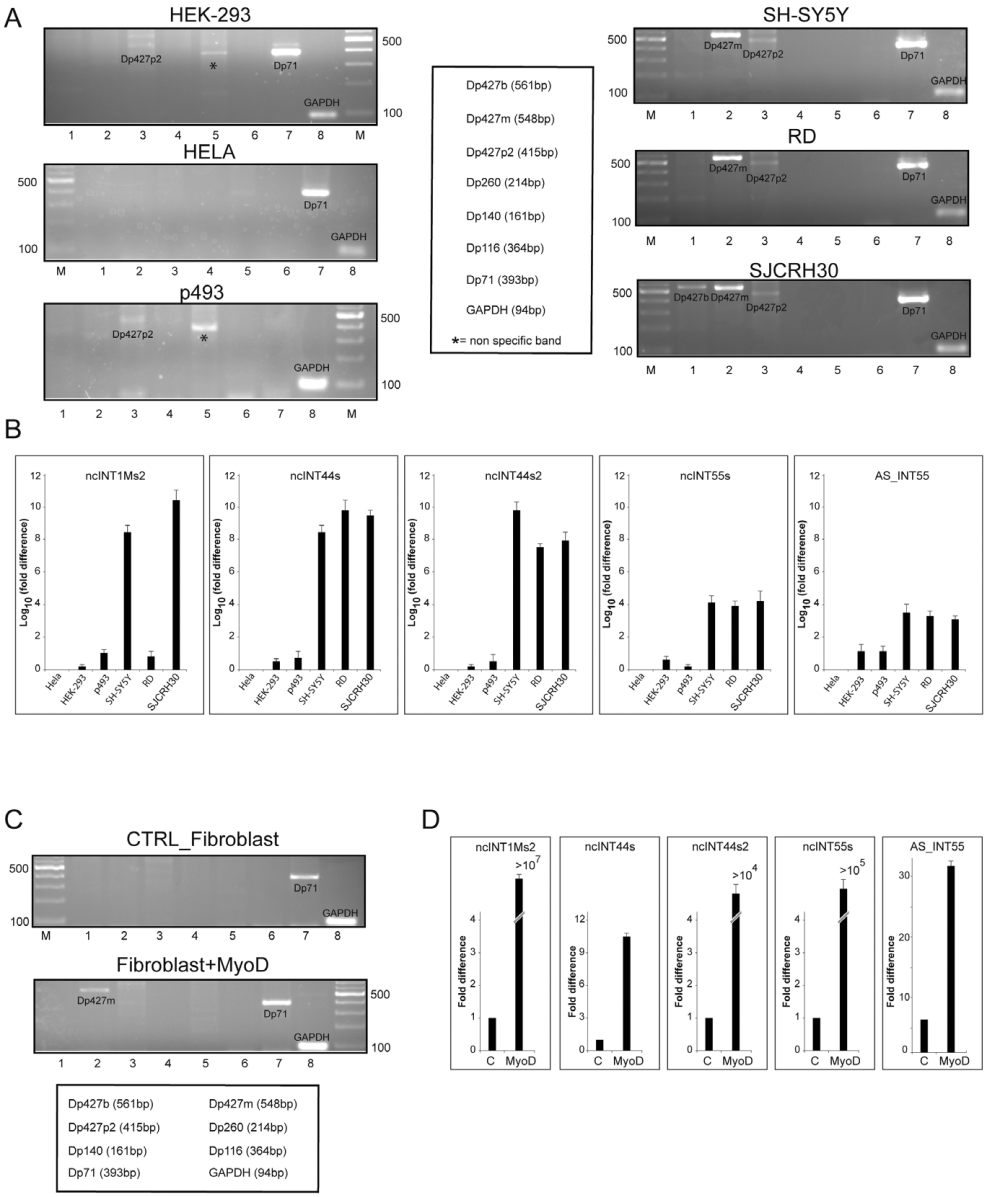
Expression of lncRNA correlates with that of full-length dystrophin isoforms. A) Standard RT-PCR of various dystrophin isoforms in six different human cell lines: HEK-293 (embryo kidney), HeLa (epithelial cervical cancer) p493 (lymphoblastoid), SH-SY5Y (neuroblastoma), RD (rhabdomyosarcoma) SJCRH30 (rhabdomyosarcoma), B) Expression of ncRNA in different cell lines determined by qRT-PCR. HeLa cells were chosen as reference cells since expression of lncRNAs in those cells was the lowest detected. Therefore, expression of lncRNAs in other cell lines was compared to that of HeLa cells, which was set to 1. Fold difference is expressed as Log_10_ of the ratio. Data represent the average of three independent experiments performed in triplicate. Standard error deviation is shown. C) Expression of dystrophin isoforms in human normal fibroblast (CTRL) and fibroblasts transformed with MyoD as determined by standard RT-PCR. D) Relative expression of lncRNA in MyoD fibroblasts (MyoD), as compared to that of untransformed fibroblasts (control, CTRL), which was set to 1. Data are the average of three independent experiments performed in triplicate. Standard error deviation is shown.

To understand whether the ncRNA expression might be functionally associated with that of dystrophin, we compared the expression of the validated ncRNAs and dystrophin isoforms in six different human cell lines. Three cell lines HEK-293, HeLa and p493 were chosen for the absence of expression of the muscle and brain dystrophin isoforms, though they express a low level of the Purkinje isoform and moderate-good levels of the Dp71 ubiquitous isoform, (Fig. 5A left). In contrast, the SH-SY5Y neuroblastoma cell line and, in particular, the two rhabdomyosarcoma cell lines (RD and SJCRH30) were chosen for their good expression of the muscle and brain dystrophin isoforms (Fig. 5A, right). Expression of five ncRNAs was monitored in the cell lines by qRT-PCR. Instead, nc3UTRas was not included in this part of the study since analysis could have been complicated by the fact that it overlaps the 3′UTR region of all full-length and 3′ dystrophin isoforms. Expression of each ncRNA was calculated as the ratio between expression of the ncRNA in a given cell line and that in HeLa cells, which were found to have the lowest level of transcription of all tested ncRNAs. As shown in Fig. 5B, expression of the five ncRNAs is several orders of magnitude higher in cells displaying good levels of full-length muscle and brain dystrophin isoforms than in the other cell lines, thereby suggesting that ncRNAs and dystrophin expression may follow similar dynamics. To further support this idea, we determined the expression levels of our ncRNAs in human normal fibroblasts and human fibroblasts previously transformed with MyoD, a well recognised master transcription regulator that can induce fibroblasts to undergo myogenesis [55]. Results show that MyoD-transformed fibroblasts present strong expression of all tested ncRNAs, as compared to untransformed fibroblasts, which parallels the expression of full-length muscle and brain DMD isoforms.

RT-PCR crossing the regions where the DMD ncRNAs are transcribed amplified only the normal dystrophin junctions, thereby demonstrating that these ncRNAs are not spliced within the full-length DMD transcript (data not shown).

### DMD lncRNAs are Confined to the Nuclear Compartment

**Figure 6:**
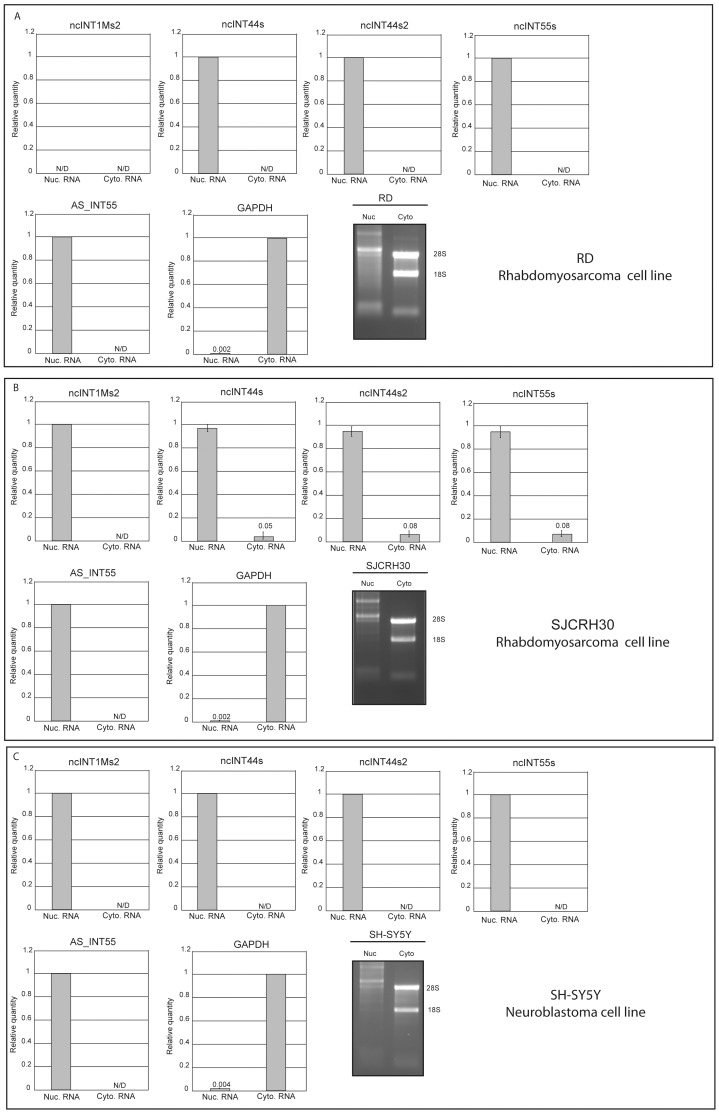
Intracellular localization of lncRNAs in three distinct human cell lines. Cytoplasmic and nuclear fractions of cellular RNA were analysed for lncRNA expression by qRT-PCR. Cyt = Cytoplasm, Nuc = Nuclear. qRT-PCR was performed for more than 50 cycles in order to detect even extremely low transcript levels. When expression was detected just in one compartment, the relative quantity of a given lncRNA was set to 1, indicating that 100% of the transcript was observed only in that compartment. In contrast, when both compartments expressed some levels of the lncRNA, distribution of the transcript between the two compartments was quantified as a relative percentage. In the specific case, standard error deviation was calculated. Quality of extracted RNAs was evaluated by running 1 ug of nuclear and cytoplasmic RNAs on a denaturing agarose gel. 28S and 18S ribosomal RNA are indicated as molecular weight markers.

To further explore whether analysed ncRNAs may be functionally linked to the expression dynamics of the DMD locus, their intracellular distribution was determined. GAPDH mRNA was used as a fractionation control, since it is known to be restricted to the cytoplasm. Results show that all tested ncRNAs are essentially nuclear (Fig. 6), suggesting that expression of the ncRNAs may be important for the transcriptional architecture of the DMD locus.

### Sense ncRNAs Negatively Modulate Dp427m, Dp427b and Dp427p Isoforms but not Dp71

To investigate the function of our long ncRNAs, we reasoned that similarly to other long ncRNAs function, DMD ncRNAs may act to regulate the expression of the dystrophin transcription. To test this hypothesis, we initially used RNA interference to deplete the expression of a ncRNAs. However, this approach failed, most likely because siRNAs cannot efficiently target nuclear transcripts (Methods S1 and Figure S4).

**Figure 7:**
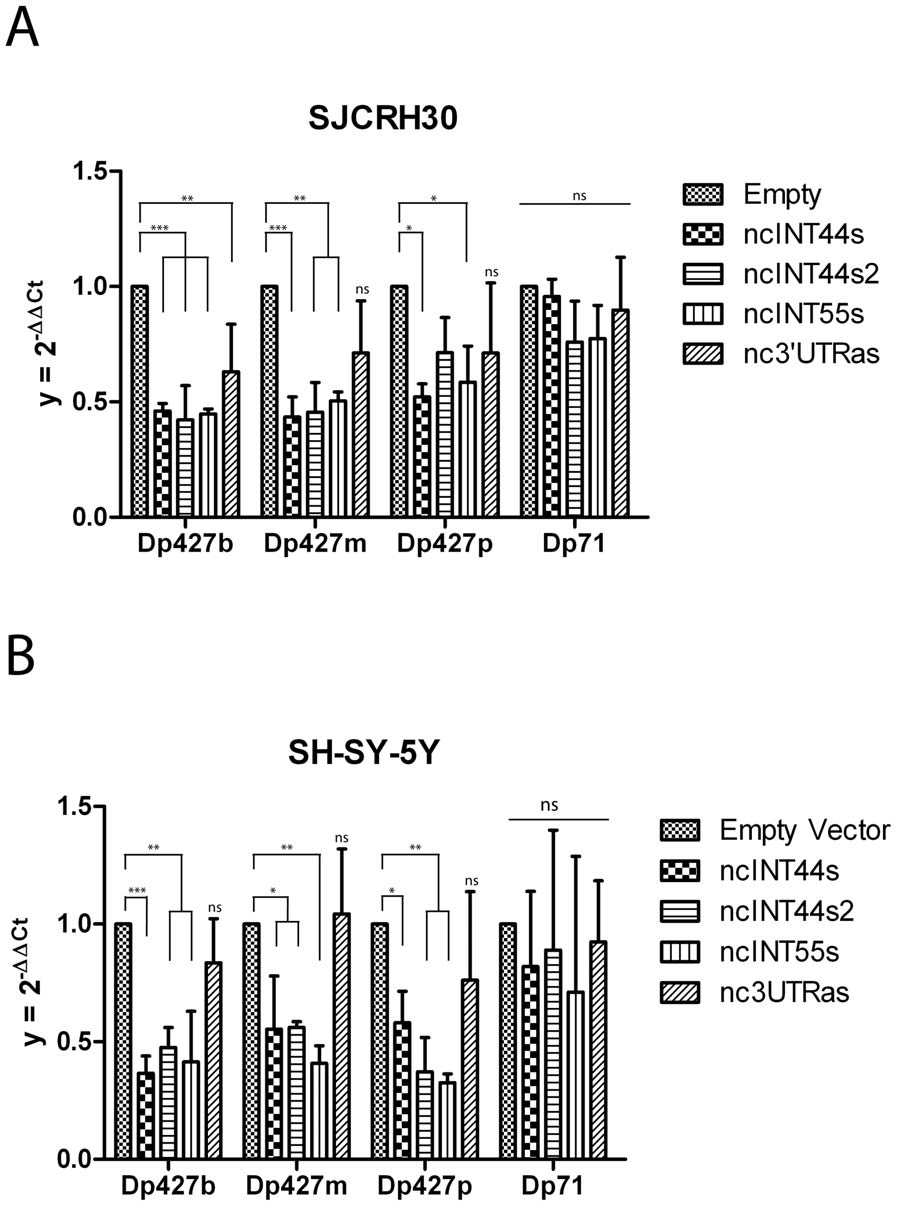
Overexpression of individual lncRNAs downregulates muscle and brain dystrophin mRNA isoform expression. SJCRH30 (panel A) and SH-SY-5Y (panel B) cells were separately transfected with vectors expressing ncINT44s, ncINT44s2, ncINT55s, nc3UTRas respectively. Endogenous expression of Dp427b, Dp427m, Dp427p and Dp71 dystrophin mRNA isoforms was determined by qRT-PCR with specific TaqMan systems. The pcDNA3.1(+) empty vector was used as a negative control. Amount of each dystrophin isoform was normalized to ß-actin mRNA, and was expressed as 2^-ΔΔCt^. Dystrophin expression levels determined for each ncRNA transfection were compared to the condition with the empty vector which was set to 1. Statistical analyses were performed using one-way ANOVA with Dunnett’s test based on results of three indepedendent transfections in which each transfection point was performed in triplicate. The error bars and asterisks represent the standard deviations and p-values respectively. (*) =  p<0.05; (**) p< = 0.01; (***) = p<0.001, ns = non significant.

We decided then to adopt an alternative approach through which we wished to perturbate the endogenous dystrophin expression by exogenous overexpression of the DMD lncRNAs. To this purpose ncINT44s, ncINT44s2, ncINT55s and nc3UTRas sequences were cloned into the pcDNA3.1(+) expression vector, under the transcriptional control of the CMV promoter. Rhabdomyosarcoma (SJCRH30) or neuroblastoma (SH-SY-5Y) cells were individually transfected with each ncRNA. As a negative control, cells were also transfected with the pcDNA3.1(+) empty vector. Total RNAs were prepared and tested by qRT-PCR for expression levels of endogenous dystrophin isoform mRNAs. GraphPad Prism 5 software and one-way ANOVA with Dunnett’s test, were used to statistically analyse the mean of RT-PCR results obtained from triplicates of three independent experiments performed in each tested cell line. Figure 7, shows that, with the sole exception of the nc3UTRas, overexpression of each long non coding transcript results in a significant reduction of Dp427m and Dp427b in both cell lines when compared to the control sample. Although modest we could also observe some reduction on the Purkinje dystrophin isoform (Dp427p). Importantly, no variation was observed with regard to the Dp71 transcript, suggesting that the effect of sense lncRNAs on muscle and brain dystrophin isoforms is specific. Overall our finding support a model in which specific DMD lncRNAs can control muscle and brain dystrophin isoforms by down-modulating their transcription level.

**Figure 8:**
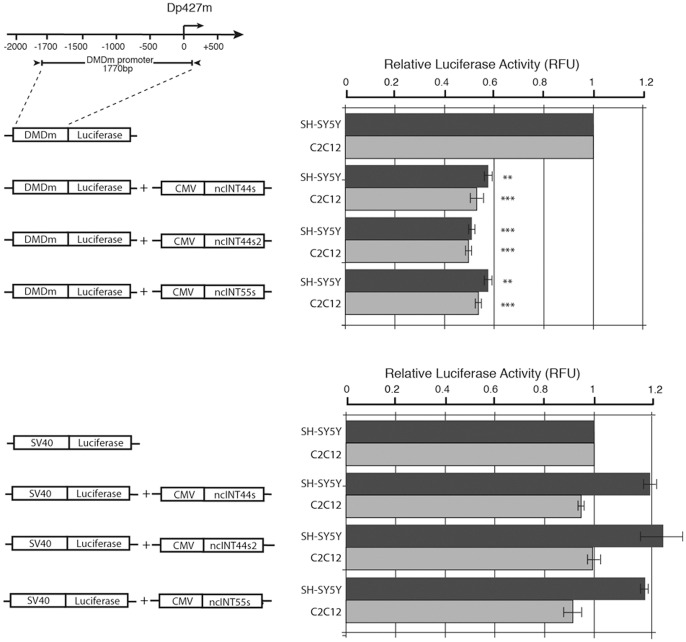
lncRNAs down-regulate expression of a luciferase reporter driven by the Dp427m promoter. Cartoons describes the Luc-reporter construct and lncRNAs expression vectors. Expression of the muscle dystrophin reporter was determined as a function of co-transfection of the ncNT44s, ncNT442s and ncNT55s RNAs and of the expression vector alone used as the negative control whose activity was arbitrarily set to 1. An SV40-luc reporter was used as a control of the specificity of the lncRNA effect. Transfections were performed in human neuroblastoma SH-SY5Y cells (drak grey bars) as well as in mouse C2C12 myoblasts (light grey bars). Results are the average of four indipendent trasfection experiments in which each point was analysed in triplicate. Statistical significance is indicated by either two or three asterisks representing p-values <0.01 and 0.001 respectively.

Finally to understand how lncRNAs can affect dytrophin transcription we have analysed their effect on a luciferase reporter gene whose expression is driven by the Dp427m promoter. A SV40-luc reporter was also used as a negative control. The analysis was performed both in SH-SY5Y human cells and in mouse C2C12 undifferentiated myoblasts. Figure 8 shows that all three lncRNAs can significantly down-regulate expression of the Dp427m-Luc reporter but not that of the SV40-luc reporter, thus supporting the model that DMD lncRNAs can negatively control muscle specific dystrophin isoform expression by targeting the promoter.

### Relative Expression of lncRNAs and Dystrophin Isoforms in Muscles of DMD Female Carriers

To support the idea that a negative relationship between lncRNAs and dystrophyn mRNA levels may exist, we analysed expression of lncRNAs in muscle samples of 9 DMD female carriers either healthy or mildly affected (Table 1). Since the relative amount of dystrophin mRNA can be significantly different among samples, the relative amount of each lncRNAs was compared with the ratio between dystrophin mRNA and lncRNAs amounts of the same sample. Through this mathematical device we should be able to observe whether the two variables (ncRNA and dystrophin mRNA) are somehow causally linked or completely independent.

**Figure 9:**
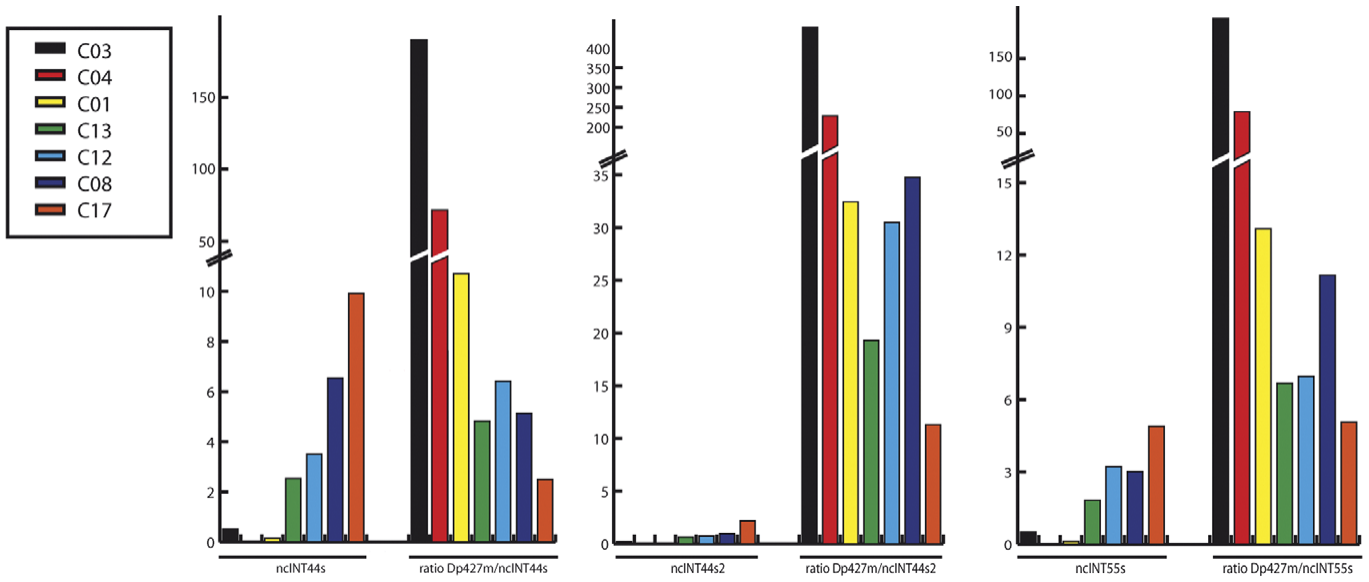
Correlation between the expression of different dystrophin mRNA isoforms and expression of ncRNAs. Expression of individual ncRNAs as well as of distinct dystrophin isoforms was determined in muscle samples obtained from DMD female carriers by qRT-PCR. Relative amount of each lncRNA was correlated to the the ratio between dystrophin mRNA amount and lncRNAs amount. In the graph female carriers were ordered based on their relative expression of lncRNAs ranging from that with lowest to that with highest lncRNA levels. The same order was maintained for dystrophin/lncRNA ratios.

As predicted we found an inverse correlation between ncRNA expression and Dystrophin expression existed (Fig. 9), thus supporting the model of a negative regulation of lncRNAs on specific muscle dystrophin transcripts.

## Discussion

The dystrophin gene is an extraordinary source of information for unravelling molecular mechanisms underlining transcription regulation in humans, as demonstrated by the extreme fine-tuning of dystrophin transcription, its correlation with specific phenotypes and basic mechanisms, and the recent molecular therapy approach based on splicing modulation [68].

Taking all this into account, exploration of the occurrence of regulatory RNAs within this gene represents an appealing and obvious target, and their full characterisation will surely have great impact on our understanding of the genetic programming of the DMD locus.

In this context, the development of our gene-specific Gene Expression microarray has led for the first time to the definition of the contribution of the *DMD* gene to non-coding RNA production. In fact, we could identify 14 non-coding poly A+ transcripts in human adult skeletal muscle, brain, heart and skin tissues transcribed mostly within introns and sense-oriented like dystrophin transcripts.

Tiling arrays of the entire human genome have expanded upon these analyses, detecting messages in the liver that map 1,529 and 1,566 novel intronic transcriptionally-active regions, arising, respectively, from the antisense or sense strands of the corresponding genes [69]. Although several transcribed regions have previously been identified in whole chromosomes, including the X chromosome, in a similar fashion, the *DMD* gene has never before been examined in such a detail in human skeletal muscle, brain or heart tissues. By doing so, our approach, has allowed the discovery of completely new transcripts for this genomic region that were not identified by previous array experiments, thereby demonstrating the sensitivity of our method. Further investigations demonstrated that the identified transcripts are mostly tissue-specific, highly expressed during myogenesis, and compartmentalised into the nucleus.

### Architecture, Compartmentalisation and Conservation of Long Non-coding RNAs in the DMD Gene

ncRNAs are currently categorised on the basis of their structure, and the transcripts we identified show several structural features common to many previously characterised lncRNAs.

In general, as reported for other non-coding transcripts [43], our transcripts are poorly conserved. Sequence conservation could be relevant for lncRNAs likely to function in trans, where secondary structure is a requirement for the recognition of RNA-binding protein targets, and in order to exert the specific cellular functions. In fact, lncRNA expression seems to be subject to diverse levels of evolutionary constraint in mammals. In general, small ncRNAs such as miRNAs are highly conserved, while longer transcripts are less prone to conservation than typical exons. Xist, responsible for guiding X chromosome inactivation, and Air, involved in mouse-imprinted gene silencing at the Igf2r locus, are examples of poorly conserved lncRNAs. Basing on these observations, as well as on our functional data (see below), our DMD lncRNAs seem to belong to this latter class of ncRNAs, therefore their regulatory role is expected to be in *cis*.

The length of our characterised transcripts ranged from about 1800 to 2800 nt, which is well above the typical length reported for exons of protein-coding genes (mean 141 nt). As for several other lncRNAs [70]–[73], two of our characterised lncRNAs also exist in different isoforms, which are differentially expressed in the tissues studied, presumably reflecting tissue-specific functions.

Moreover, with the exception of ncINT55as, most identified ncRNAs are unspliced, similar to Kcnq1ot1 [74] and MENepsilon/beta isoforms [71]. Unbiased analyses have already indicated that a significant proportion of un-annotated ncRNAs are exclusively detected in nuclear or cytoplasmic cellular extracts [70], [75]. Intronic ncRNA expression seems to be predominantly nuclear, although some subsets have primarily been detected in the cytoplasm, and only a few seem to be equally expressed in both compartments [76]. Our results are consistent with these observations, since all analysed transcripts were found to be mainly, if not exclusively, located in the nuclear compartment.

### DMD lncRNAs Function

lncRNAs are known to be involved in three main functional areas: i) chromatin modification and epigenetic regulation; ii) subcellular and structural organisation of transcripts; and iii) regulation of expression of neighbouring genes either in cis or in trans (a phenomenon known as *transvection*) [11]. Our transcripts were observed to have preferential distribution in proximity to the unique first exons of several DMD gene isoforms, as reported for other ncRNAs [69], [76]. Interestingly, considering the full repertoire of human lncRNAs, a higher proportion of transcripts are mapped to the first introns of protein-coding genes, a region relatively close to the promoters. This intense transcription activity of ncRNAs around promoter regions appears to relax the chromatin structure, making it more accessible to the transcription machinery [35]. Nevertheless, a contrasting phenomenon also appears to hold some truth. For instance, a partially intronic ncRNA produced from the genome locus that encodes dihydrofolate reductase (*DHFR*) directly interacts with the DHFR major promoter to reduce the expression of the protein-coding RNA [27]. Along with the latter line of evidence stand our data. Indeed we have shown that forced expression of specific lncRNAs in human cells can down-regulate expression of muscle and brain dystrophin isoforms. Apparently this negative modulation is achieved through some kind of interaction between the lncRNA and the dystrophin promoter region. Although additional work is clearly required to characterize this mechanism and to identify the cellular components involved in this phenomenon, nonetheless our analyses have revealed that some DMD lncRNAs can specifically target the dystrophin promoter and somehow dictate the dystrophin transcription rate. This is also corroborated by the analyses performed on muscle samples of DMD female carriers and from indirect observations that, for instance, a lncRNA like ncINT1Ms2, whose 3′ end overlaps the promoter region of the Dp427p full-length dystrophin isoform, is expressed in the heart and in the skeletal muscle where Dp427p is not expressed, whereas it is undetectable in the brain where Dp427p is produced.

Despite their relevance for the biology of the dystrophin gene, our data become particularly significant for a more comprehensive understanding of dystrophinopathies and eventually for the better design of the therapeutic approaches. For instance a similar scenario to that unravelled by this study has been recently highlighted in FSHD, in which ncRNAs were demonstrated to act as repressors of the binding copy number variations within the FSHD D4Z4 repeats with implications for the transcriptional control of putatively in cis pathogenic genes such as FRG1 [40]. Moreover, in another study it has been discovered that a particular lncRNA interacting with two miRNAs (one of which was enriched in the serum of DMD patients and could be correlated with the clinical assessment of the pathology [77]) can regulate muscle differentiation [78], with a plausible impact on the pathogenesis of DMD.

### Conclusions

In conclusion, our findings have revealed the presence of a large class of novel lncRNAs specifically transcribed by the DMD locus that are located in the nucleus and are likely to be involved in the transcriptional orchestration of many dystrophin isoforms. This study has great implications, not only in the elucidation of the physiological role of dystrophin in cells, but also in the investigation of dystrophinopathies and their pathogenesis. These data open novel investigation perspectives, as possible dynamic transcriptome signature for monitoring disease progression and therapeutic responses. It is, in fact, widely recognised that high dystrophin transcription levels are fundamental for yielding a sufficient amount of protein to be therapeutic [79], [80]. Therefore, better elucidation of factors influencing the dystrophin gene transcription is of prime importance, and may also improve recently adopted splicing modulation therapies.

## Supporting Information

Figure S1 **Northern blot analysis on a 12-lane human poly A+ RNA filter of transcripts originating near the DMD isoform first exons.** Transcripts ncINT1Ms, ncINT1Ps and ncINT29s are located near the first exon of DMD gene isoforms Dp427m, Dp427p and Dp260, respectively. Northern blot analysis of these sequences yielded smeared signals in specific lanes, suggesting that repeated sequences within these transcripts are expressed in the tissues examined.
(DOCX)

Figure S2 **Coding Potential Calculator.** The CPC on-line tool was used to detect open reading frames in our ncRNAs. None presented coding potential except for ncINT1Ms2, which showed weak results. ncINT55as and ncINT1Ms2 gave several matches when compared to 5′ and 3′ UTRs of known genes (File S1).
(DOCX)

Figure S3 **Interspecies conservation analysis using UCSC Genome Browser: all transcripts displayed poor conservation, with the exception of nc3UTRas.** ncINT1Ms2 was found to possess a small region of about 130 bp with a high LOD score.
(DOCX)

Figure S4 **Interference analysis of ncINT44s: cells transfected with siGLO green indicator served as meter of optimal transfection parameters (A).** siGLO green is composed by fluorescent oligonucleotides that localize to the nucleus (white arrows) thus permitting unambiguous visual assessment of uptake into mammalian cells. These reagents are not intended to provide information about siRNA function, localization or duration of silencing, however they are ideal defining for optimal transfection conditions for siRNA. The relative quantification of the GAPDH and ncINT44s transcripts was assessed by Real Time PCR using the ΔΔCT Method and results were displayed by using the RQ manager software (Applied Biosystem) as log10 (B).
(DOCX)

Table S1 **Accession numbers and names of genes used as controls in the custom-designed gene expression microarrays.**
(DOCX)

Table S2 **Name, number of probes and reiteration of each probe set within the 4×44k sense and antisense DMD gene expression microarrays.**
(DOCX)

Table S3 **Name and nucleotide sequence of the primers used for 5′ and 3′ RACE.**
(DOCX)

Table S4 **Name and sequence of the Taqman RealTime systems used for the compartmentalisation study.**
(DOCX)

Table S5 **Name and sequence of the primers used to amplify the DMD gene isoforms in cDNA samples.**
(DOCX)

Table S6 **Name and sequence of primers used for DMD ncRNAs cloning into pcDNA3.1(+).**
(DOCX)

Method S1 **Interference analysis of ncINT44s.**
(DOCX)

File S1 **CPC generates Blast results by aligning the ncRNAs to 5′ and 3′ UTRs of all known genes.** All the matches for ncINT1Ms2 and ncINT55as are shown.
(DOCX)

File S2 **Full FASTA sequences of the DMD lncRNAs completely characterized.**
(DOCX)
