# Supplementary figures and images for: The DMD Locus Harbours Multiple Long Non-Coding RNAs Which Orchestrate and Control Transcription of Muscle Dystrophin mRNA Isoforms
Source: PLoS One. 2012 Sep 21;7(9):e45328. doi: 10.1371/journal.pone.0045328 (PMC3448672; doi:10.1371/journal.pone.0045328)

**Figure S1**


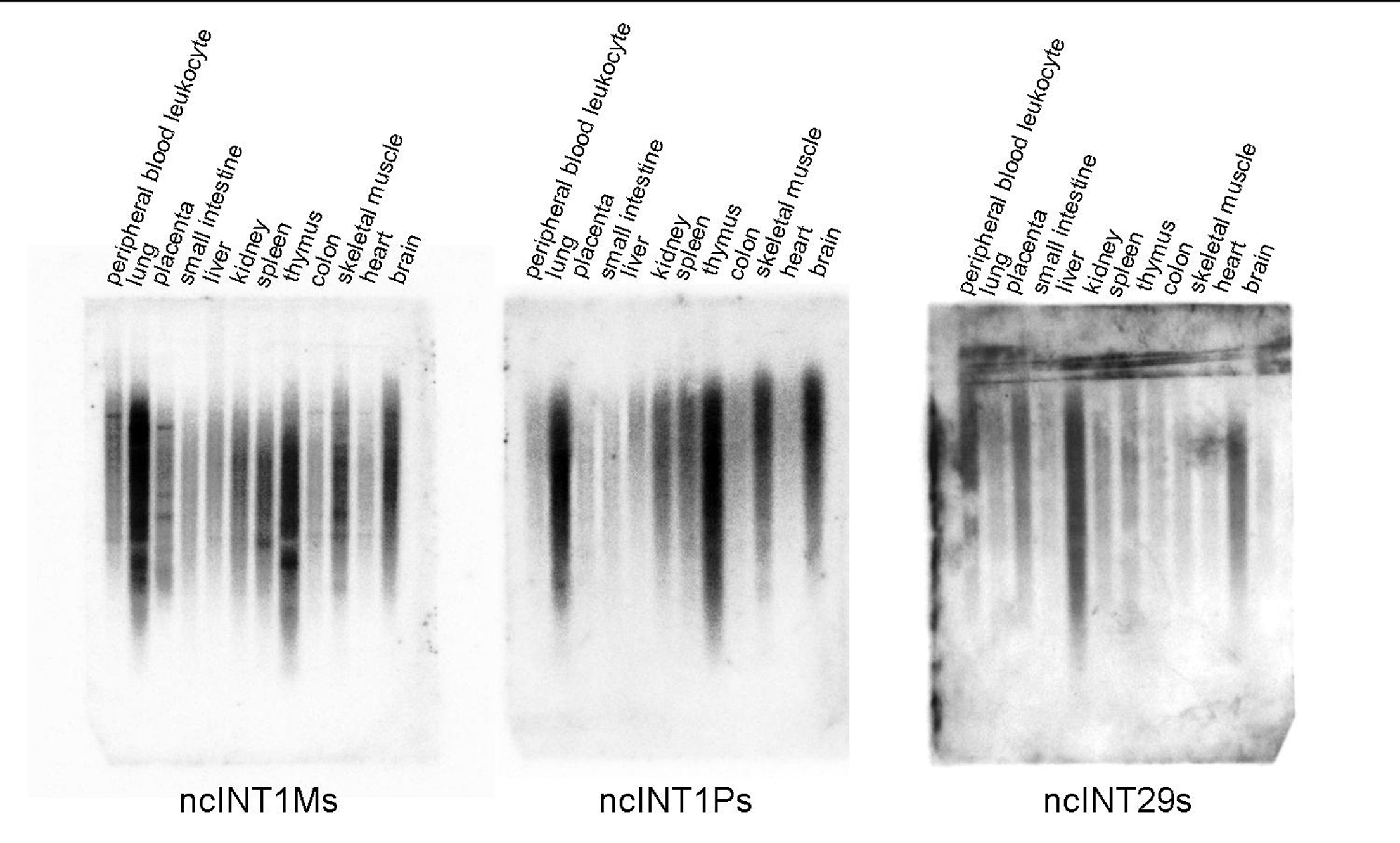

Supplement: Figure S1 — Northern blot analysis on a 12-lane human poly A+ RNA filter of transcripts originating near the DMD isoform first exons. Transcripts ncINT1Ms, ncINT1Ps and ncINT29s are located near the first exon of DMD gene isoforms Dp427m, Dp427p and Dp260, respectively. Northern blot analysis of these sequences yielded smeared signals in specific lanes, suggesting that repeated sequences within these transcripts are expressed in the tissues examined. (DOCX) [file pone.0045328.s001.docx]

**Figure S2**


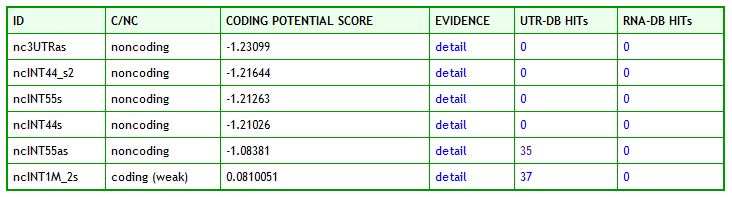

Supplement: Figure S2 — Coding Potential Calculator. The CPC on-line tool was used to detect open reading frames in our ncRNAs. None presented coding potential except for ncINT1Ms2, which showed weak results. ncINT55as and ncINT1Ms2 gave several matches when compared to 5′ and 3′ UTRs of known genes (File S1). (DOCX) [file pone.0045328.s002.docx]

Figure S3
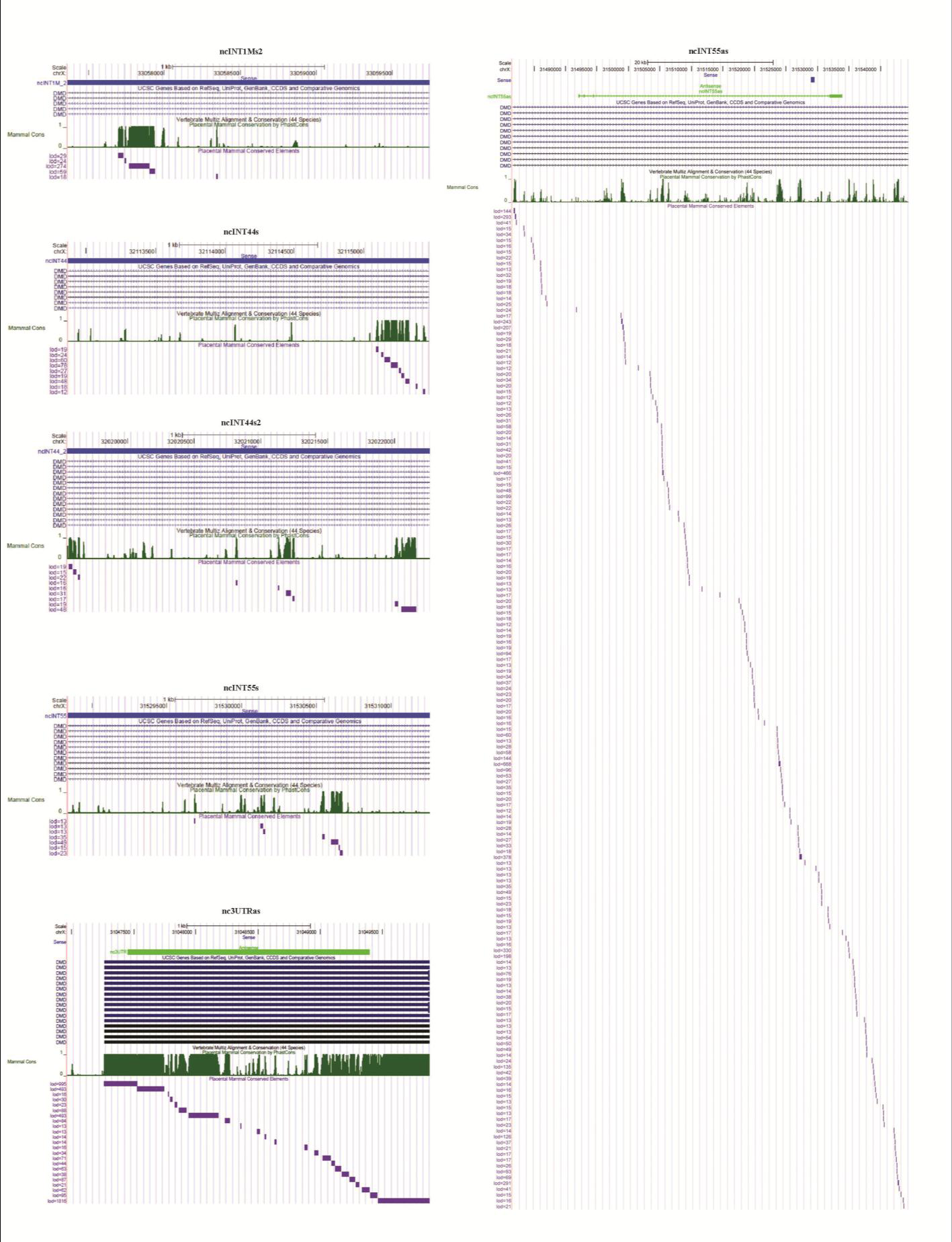

Supplement: Figure S3 — Interspecies conservation analysis using UCSC Genome Browser: all transcripts displayed poor conservation, with the exception of nc3UTRas. ncINT1Ms2 was found to possess a small region of about 130 bp with a high LOD score. (DOCX) [file pone.0045328.s003.docx]

**Figure S4**


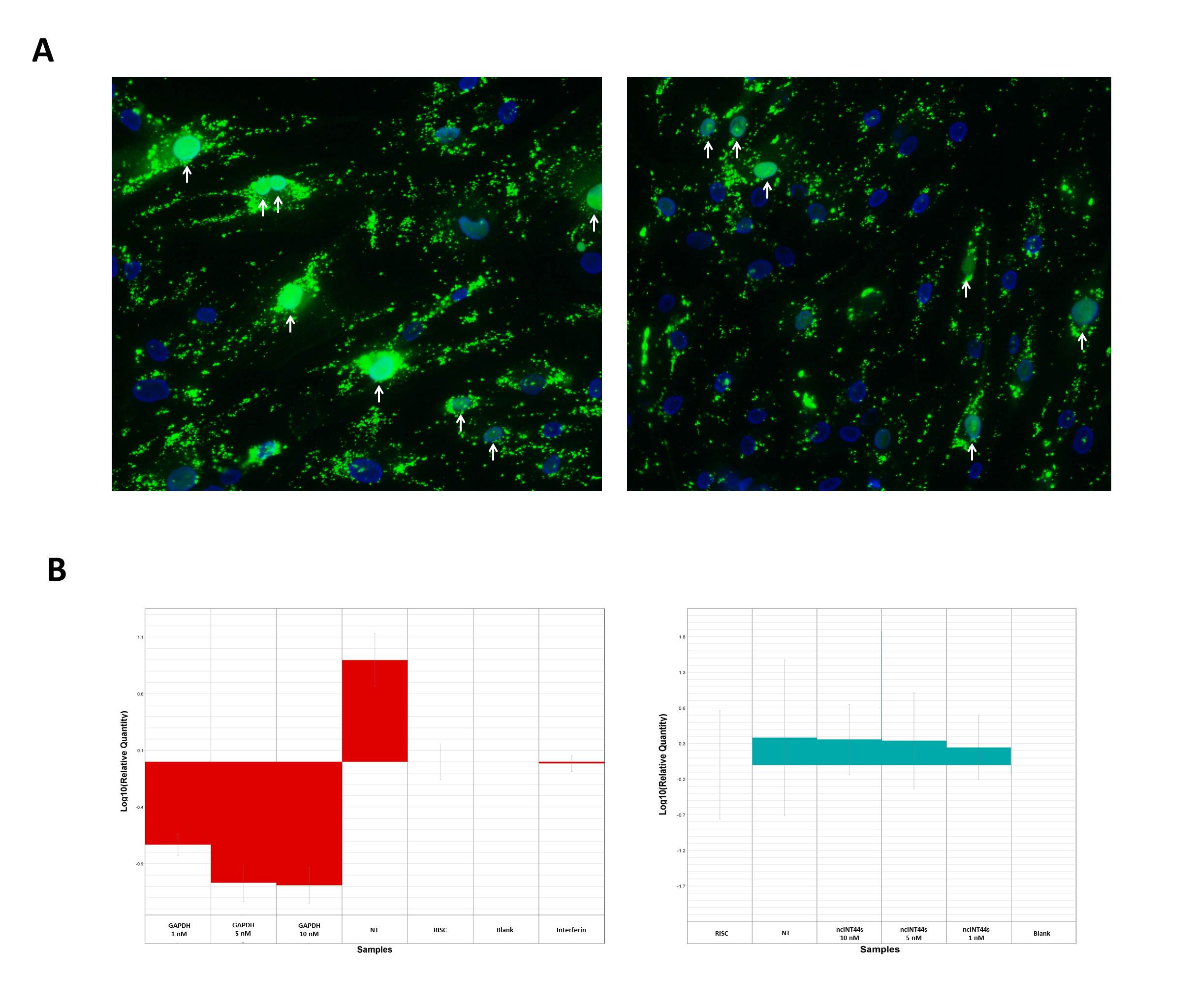

Supplement: Figure S4 — Interference analysis of ncINT44s: cells transfected with siGLO green indicator served as meter of optimal transfection parameters (A). siGLO green is composed by fluorescent oligonucleotides that localize to the nucleus (white arrows) thus permitting unambiguous visual assessment of uptake into mammalian cells. These reagents are not intended to provide information about siRNA function, localization or duration of silencing, however they are ideal defining for optimal transfection conditions for siRNA. The relative quantification of the GAPDH and ncINT44s transcripts was assessed by Real Time PCR using the ΔΔCT Method and results were displayed by using the RQ manager software (Applied Biosystem) as log10 (B). (DOCX) [file pone.0045328.s004.docx]
