# Supplementary material for: The DMD Locus Harbours Multiple Long Non-Coding RNAs Which Orchestrate and Control Transcription of Muscle Dystrophin mRNA Isoforms
Source: PLoS One. 2012 Sep 21;7(9):e45328. doi: 10.1371/journal.pone.0045328 (PMC3448672; doi:10.1371/journal.pone.0045328)
Supplement: Table S1 — Accession numbers and names of genes used as controls in the custom-designed gene expression microarrays. (DOCX) [file pone.0045328.s005.docx]

**Table S1**

Accession numbers and names of genes used as controls in the custom-designed DMD-GEx

microarrays.

| **Accession Number** | **Transcript Name** |
| --- | --- |
| NM_018955.2 | Ubiquitin B (UBB) |
| NM_053275.3 | Ribosomal protein, large, P0 (RPLP0) |
| NM_000975.2 | Ribosomal protein L11 (RPL11) |
| HSU39317 | Ubiquitin-conjugating enzyme UbcH5B (UBCH5B) |
| NM_013442.1 | Stomatin (EPB72)-like 2 (STOML2) |
| NM_012100.1 | Aspartyl aminopeptidase (DNPEP) |
| NM_002803.2 | Proteasome 26S subunit, ATPase, 2 (PSMC2) |
| NM_015345.2 | Dishevelled associated activator of morphogenesis 2 (DAAM2) |
| NM_012106.3 | ADP-ribosylation factor-like 2 binding protein (ARL2BP) |
| NM_015024.2 | Exportin 7 (XPO7) |
| NM_012179.3 | F-box protein 7 (FBXO7) |
| NM_181805.1 | Protein kinase (cAMP-dependent, catalytic) inhibitor gamma (PKIG) |
| NM_021959.2 | Protein phosphatase 1, regulatory (inhibitor) subunit 11 (PPP1R11) |
| NM_001722.2 | Polymerase (RNA) III (DNA directed) polypeptide D, (POLR3D) |
| NM_004737.3 | Like-glycosyltransferase (LARGE) |
| NM_004748.3 | Cell cycle progression 1 (CCPG1) |
| NM_003047.2 | Solute carrier family 9 (sodium/hydrogen exchanger)(SLC9A1) |
| NM_004045.3 | ATX1 antioxidant protein 1 homologue (ATOX1) |
| NM_002766.1 | Pyrophosphate synthetase-associated protein 1 (PRPSAP1) |
| NM_001428.2 | Enolase 1, (alpha) (ENO1) |
| NM_003095.2 | Small nuclear ribonucleoprotein polypeptide F (SNRPF) |
| NM_001914.2 | Cytochrome b5 type A (microsomal) (CYB5A) |
| NM_000426.3 | Laminin, alpha 2 (merosin, congenital muscular dystrophy) (LAMA2) |
| NM_003279.2 | Troponin C type 2 (fast) (TNNC2) |
| NM_003280.1 | Troponin C type 1 (slow) (TNNC1) |
| NM_003281.3 | Troponin I type 1 (skeletal, slow) (TNNI1) |
| NM_001101.2 | Actin, beta (ACTB), |
| NM_002046.3 | Glyceraldehyde-3-phosphate dehydrogenase (GAPDH) |
| NM_006275.4 | Splicing factor, arginine/serine-rich 6 (SFRS6) |
| NM_005626.3 | Splicing factor, arginine/serine-rich 4 (SFRS4) |
