# Supplementary material for: The DMD Locus Harbours Multiple Long Non-Coding RNAs Which Orchestrate and Control Transcription of Muscle Dystrophin mRNA Isoforms
Source: PLoS One. 2012 Sep 21;7(9):e45328. doi: 10.1371/journal.pone.0045328 (PMC3448672; doi:10.1371/journal.pone.0045328)
Supplement: Table S2 — Name, number of probes and reiteration of each probe set within the 4×44k sense and antisense DMD gene expression microarrays. (DOCX) [file pone.0045328.s006.docx]

**Table S2**

| **Probe set name** | **N° of probes** | **Replicated in DMD GEx sense** | **Replicated in DMD GEx antisense** |
| --- | --- | --- | --- |
| Sense DMD | 14928 | 2 | 0 |
| Sense DMD exons | 174 | 8 | 8 |
| Sense DMD CNS | 2411 | 3 | 0 |
| Sense DMD MiR | 51 | 9 | 0 |
| Sense DMD promoters | 34 | 8 | 0 |
| Sense Controls | 295 | 8 | 5 |
| Antisense DMD | 14914 | 0 | 2 |
| Antisense DMD CNS | 2421 | 0 | 3 |
| Antisense DMD exons | 169 | 4 | 3 |
| Antisense DMD promoters | 53 | 0 | 8 |
| Antisense DMD MiR | 51 | 0 | 8 |
| Antisense Controls | 311 | 5 | 8 |
|  | **Total** | **43803** | **43785** |

Name, number of probes and reiteration of each probe set within the 4x44k sense and antisense DMD gene expression microarrays.
