# Supplementary material for: The DMD Locus Harbours Multiple Long Non-Coding RNAs Which Orchestrate and Control Transcription of Muscle Dystrophin mRNA Isoforms
Source: PLoS One. 2012 Sep 21;7(9):e45328. doi: 10.1371/journal.pone.0045328 (PMC3448672; doi:10.1371/journal.pone.0045328)
Supplement: Table S3 — Name and nucleotide sequence of the primers used for 5′ and 3′ RACE. (DOCX) [file pone.0045328.s007.docx]

**Table S3**

| Primers used for 3’ RACE | |
| --- | --- |
| Primer | Nucleotide sequence (5’-3’) |
| T2_GSP1 | cgccctcgagctaggacatggaac |
| T2_GSP2 | tcacatttaagagggaaggagaaacgcc |
| T2_GSP3 (3’) | cccaccaacacctcctgttctctcc |
|  |  |
| 44_8GSP1 | aaatccatattcctgggggcgatgc |
| 44_8GSP2 | cgaaggtgcagccagactgggagtt |
| 44_8GSP3 (3’) | tgtgggactgcctcttgctcctgaca |
|  |  |
| 44_9GSP1 | cccagaaggggccttgtctgaagtg |
| 44_9GSP2 | ccagcggattacggtgtgggtgaat |
| 44_9GSP3 (3’) | tcagggatgctgctgctctctaggc |
|  |  |
| 55_11GSP1 | ggttatgcttcccgtctcttcacagagc |
| 55_11GSP2 | cccacgatctggaacagactggcggata |
| 55_11GSP3 (3’) | ccgcaataactctgtgaagtgctg |
|  |  |
| AS55_GSP1 | aggacatgaatggatgataatttgtggg |
| AS55_GSP2 | gaaaagcatgccataagctgttcttagc |
| AS55_GSP3 (3’) | tggagcagtgaaaagtagattttggtgtcg |
|  |  |
| AS3UTR_GSP1 | cagggatgggctgggaatccatag |
| AS3UTR _GSP2 | ggcattgctagcagcaggaagctg |
| AS3UTR _GSP3 (3’) | ctgccccactcagctgacagttctc |

| Primers used for 5’ RACE | |
| --- | --- |
| Primer | Nucleotide sequence (5’-3’) |
| T2_GSPRT | gatgcggaaatttcatttgg |
| T2_TWrev | ttagcatgccctttcaatcag |
| T2_TW1 | ccagagctaatgaggccaag |
| T2_TW2 | tgagctccaatctcctcacc |
| T2_TW3 | tcagttgattctgatgagcacc |
| T2_GSP1 | caggtgaggagattggagctcagagagg |
| T2_GSP2 | gcccctagacccgtggttctccaac |
| T2_GSP3 (5’) | aacccagtgcccaaactgcacttca |
|  |  |
| 44_8GSPRT | ccctcttttctaccttctaggc |
| 44_8TWrev | ggacaaaagggttgcacagt |
| 44_8TW1 | aggtagtccattttgcatcagg |
| 44_8TW2 | aacaaaacctccccaatgc |
| 44_8TW3 | tcattctgagaaccacatctgc |
| 44_8GSP1 | cagcttgaaatatgtgcattggggagg |
| 44_8GSP2 | ttgagtggggcaatttctggcagg |
| 44_8GSP3 (5’) | tggcaggtggggaagagagtaaagaag |
|  |  |
| 44_9GSPRT | cagttccattctccacagg |
| 44_9GSP1 | ttcatccactgatggacgtttaggttg |
| 44_9GSP2 | tagtgggattgctggatcatctggtagc |
| 44_9GSP3 (5’) | aaagcaagacaggtcaacaaaggccag |
|  |  |
| 55_11GSPRT | gttcagactatgtccatacacagg |
| 55_11TWrev | tctgtgaagagacgggaagc |
| 55_11TW1 | gggtcacaaacgtgaaggtc |
| 55_11TW2 | ccttccagattcagcatgtg |
| 55_11TW3 | tatctctaggccgtcggttc |
| 55_11GSP1 | tcctgctttctcttccttgggttgc |
| 55_11GSP2 | cagccatggcctcacataggtgg |
| 55_11GSP3 (5’) | cccgagtagctgatgtcccttcagc |
|  |  |
| AS55_GSPRT | agctacaagctgctagacagg |
| AS55_GSP1 | caaataagctgttggtggcaggaggtg |
| AS55_GSP2 | tcagcatttcccaggaagggtgatg |
| AS55_GSP3 (5’) | gggtcaagaatgcgaaggtcaaggag |
|  |  |
| AS3UTR_GSPRT | tctctgcgagtagttccacac |
| AS3UTR_GSP1 | gcttttggagagtgggctgacatcaagtg |
| AS3UTR _GSP2 | tgcacacctgagttcacagcttcacc |
| AS3UTR _GSP3 (5’) | tgacgctggaccttttctttacccaagg |
