# Supplementary material for: The DMD Locus Harbours Multiple Long Non-Coding RNAs Which Orchestrate and Control Transcription of Muscle Dystrophin mRNA Isoforms
Source: PLoS One. 2012 Sep 21;7(9):e45328. doi: 10.1371/journal.pone.0045328 (PMC3448672; doi:10.1371/journal.pone.0045328)
Supplement: Table S4 — Name and sequence of the Taqman RealTime systems used for the compartmentalisation study. (DOCX) [file pone.0045328.s008.docx]

**Table S4**

Name and sequence of the Taqman RealTime systems used for the ncRNAs compartmentalisation study and dystrophin transcripts expression levels.

| **Name** | **Sequence** |
| --- | --- |
| ncINT1Ms2_F | TTTGGATGCAGTACTCTTTGTAGACA |
| ncINT1Ms 2_R | GGAGGTGTTGGTGGGAAAAA |
| ncINT1Ms 2_Probe | 6-FAM-AATACGGTACGATTAATG |
|  |  |
| ncINT44s_F | GGCTCCCTCATTCAATGAATCTA |
| ncINT44s_R | CAGCAAAAGATGTCAGGAGCAA |
| ncINT44s_Probe | 6-FAM-ACTATGTGGGACTGCCT |
|  |  |
| ncINT44s2_F | ACCAGGAGCTCTGCTTGCAT |
| ncINT44s2_R | TTGTGCATGATAATGTGCCTCAA |
| ncINT44s2_Probe | 6-FAM-CTGGGAGTTGCCCATG |
|  |  |
| ncINT55s_F | TGATAACTTTCATGCCCATTAACATAG |
| ncINT55s_R | AACAGGACACAAATTCAGCACTTC |
| ncINT55s_Probe | 6-FAM-ACCGCAATAACTCTG |
|  |  |
| ncINT55as_F | CATTTAGAGCAAGAGATACAGGCATT |
| ncINT55as_R | GGGCACAAAAAAGAATAATTTGCTA |
| ncINT55as_Probe | 6-FAM-AATGTATTACACTGCTACTAAAG |
| Dp427b_F | TTGATTTGTTACAGCAGCCAACTT |
| Dp427b_R | CTTCCATGCCAGCTGTTTTTC |
| Dp427b_Probe | 6-FAM TGGCATGATGGAGTGACA |
|  |  |
| Dp427m_F | GAAGAACTTTTACCAGGTTTTTTTTAT |
| Dp427m_R | CTTCTTCCCACCAAAGCATTTT |
| Dp427m_Probe | 6-FAMTGCCTTGATATACACTTTT |
|  |  |
| Dp427p_F | CATAGAATGTGTAAGAGAAAAGTACCAACA |
| Dp427p_R | GCTGGCTACACACCTTCATAGGA |
| Dp427p_Probe | 6-FAM AAATCAGCAAAAAGC |
|  |  |
| Dp71_F | TGCAGCCATGAGGGAACAG |
| Dp71_R | GGATGGTCCCAGCAAGTTGT |
| Dp71_Probe | 6-FAM TCAAAGGCCACGAGACT |
|  |  |
|  | **Part Number** |
| Human ACTB | 4352035E |
|  |  |
| Human GAPDH | 4333764F |
