# Supplementary material for: The DMD Locus Harbours Multiple Long Non-Coding RNAs Which Orchestrate and Control Transcription of Muscle Dystrophin mRNA Isoforms
Source: PLoS One. 2012 Sep 21;7(9):e45328. doi: 10.1371/journal.pone.0045328 (PMC3448672; doi:10.1371/journal.pone.0045328)
Supplement: Table S5 — Name and sequence of the primers used to amplify the DMD gene isoforms in cDNA samples. (DOCX) [file pone.0045328.s009.docx]

**Table S5**

Name and sequence of the primers used to amplify the DMD gene isoforms in cDNA samples.

| **Primer** | **Sequence** |
| --- | --- |
| Dp427bFw | CGTATCAGATAGTCAGAGTGGTTAC |
| Dp427mFw | CCTGGCATCAGTTACTGTGTTGAC |
| Dp427p2Fw | CCTATGAAGGTGTGTAGCCAGCC |
| Dp427Rv | CCATCTACGATGTCAGTACTTCC |
| Dp260Fw | AGGAACATTCGACCTGAGAAAG |
| Dp260Rv | TCCACCTTGTCTGCAATATAAGC |
| Dp140Fw | ATTGCTGGCTGCTCTGAACTAA |
| Dp140Rv | CATCTGTTTTTGAGGATTGCTG |
| Dp116Fw | GGGTTTTCTCAGGATTGCTATG |
| Dp116Rv | CCGGCTTAATTCATCATCTTTC |
| Dp71Fw | GAAGCTCACTCCTCCACTCGTA |
| Dp71Rv | AGCCAGTTCAGACACATATCCAC |
| GAPDH Fw | CATGGGGAAGGTGAAGGTC |
| GAPDH Rv | AACAATATCCACTTTACCAGAGTT |
