# Supplementary material for: The DMD Locus Harbours Multiple Long Non-Coding RNAs Which Orchestrate and Control Transcription of Muscle Dystrophin mRNA Isoforms
Source: PLoS One. 2012 Sep 21;7(9):e45328. doi: 10.1371/journal.pone.0045328 (PMC3448672; doi:10.1371/journal.pone.0045328)
Supplement: Table S6 — Name and sequence of primers used for DMD ncRNAs cloning into pcDNA3.1(+). (DOCX) [file pone.0045328.s010.docx]

Table S6

Name and sequence of primers used for DMD ncRNAs cloning into pcDNA3.1(+)

| **Name** | **Sequence** |
| --- | --- |
| ncINT44s(EcoRI)_F | AGAATTCGGGATATGAGATGTTGGAAG |
| ncINT44s(EcoRI)_R | AGAATTCGTCTTGACTACAGATGTCTTTT |
| ncINT44s2(BamHI)_F | AGGATCCGCCACTAATTCTTATTGCCATTTC |
| ncINT44s2(BamHI)_R | AGGATCCAAAGGGCTGCTACAGCTATTTT |
| ncINT55s(EcoRI)_F | AGAATTCCATTTTGCTGCACAATAAACAACC |
| ncINT55s(EcoRI)_R | AGAATTCCAAGGCATTAAAATCTGCATGATG |
| nc3UTRas(EcoRI)_F | AGAATTCGGTGGTTATAAAGAACACAACACG |
| nc3UTRas(EcoRI)_R | AGAATTCGGCGTGATATCCATATGAAATTCAT |
