# Supplementary material for: The DMD Locus Harbours Multiple Long Non-Coding RNAs Which Orchestrate and Control Transcription of Muscle Dystrophin mRNA Isoforms
Source: PLoS One. 2012 Sep 21;7(9):e45328. doi: 10.1371/journal.pone.0045328 (PMC3448672; doi:10.1371/journal.pone.0045328)
Supplement: Method S1 — Interference analysis of ncINT44s. (DOCX) [file pone.0045328.s011.docx]

**Method S1: Interference analysis of ncINT44s**

Primary human myoblasts from a DMD patient with a duplication involving exon 41 and from one control were obtained from muscle biopsies (after informed consent for research purposes, Ethical Approval N. 9/2005). Cells were cultured in high-glucose DMEM (GIBCO), supplemented with 20% fetal bovine serum (FBS; GIBCO) and antibiotic/antimicotic solution (Sigma).

For transfection, cells were seeded in 24-well plate and incubated with differentiation medium (2% FBS) for 2-5 days. Myotubes were transfected with siRNA duplexes (1 nM, 5nM and 10nM), ncINT44s, siGENOME GAPD control reagent (Dharmacon) and siGENOME RISC-Free control siRNA (Dharmacon), in the presence of INTERFERin transfection reagent (Polyplus Transfection) according to manufacturer’s instructions. Cells were collected 48 hours post-transfection.

To monitor cell uptake, cells, grown onto coverslips, were transfected also with siGLO green transfection indicator (Dharmacon), fixed in -20°C 8% paraformaldehyde 24 hours post-transfection and observed with Nikon Eclipse 80i fluorescence microscope (Supplementary Figure S4 A).

To test the effective interference of GAPDH and ncINT44s expression, the levels of these transcripts was assessed by Real Time PCR using the commercialy available TaqMan expression assay for GAPDH Endogenous Control and a custom Taqman assay designed with Primer Express for the ncINT44s transcript (Applied Biosystem). Total RNA from cells harvested at 48 hours was isolated using the RNeasy Kit (Qiagen) following the manufacturer’s instructions and reverse transcribed by using High Capacity cDNA Reverse Transcription Kit (Applied Biosystem).

Real-time PCR was performed in triplicate on the Applied Biosystems Prism 7300 system, using 10 ng of cDNA and default parameters. For relative quantification the ΔΔCT Method (Applied Biosystems User Bullettin #2) was utilized and results were displayed by using the RQ manager software (Applied Biosystem) as log10 (Supplementary Figure S4 B).
