# Supplementary material for: The DMD Locus Harbours Multiple Long Non-Coding RNAs Which Orchestrate and Control Transcription of Muscle Dystrophin mRNA Isoforms
Source: PLoS One. 2012 Sep 21;7(9):e45328. doi: 10.1371/journal.pone.0045328 (PMC3448672; doi:10.1371/journal.pone.0045328)
Supplement: File S1 — CPC generates Blast results by aligning the ncRNAs to 5′ and 3′ UTRs of all known genes. All the matches for ncINT1Ms2 and ncINT55as are shown. (DOCX) [file pone.0045328.s012.docx]

**File S1**

**BLAST RESULTS**

Reference: Altschul, Stephen F., Thomas L. Madden, Alejandro A. Schaffer,

Jinghui Zhang, Zheng Zhang, Webb Miller, and David J. Lipman (1997),

"Gapped BLAST and PSI-BLAST: a new generation of protein database search

programs", Nucleic Acids Res. 25:3389-3402.

Query= ncINT1Ms2

Database: UTRef all

463,416 sequences; 192,534,142 total letters

3HSAR025295 CR099964 3'UTR in Homo sapiens baculoviral IAP repeat-containing 4

5HSAR057161 BR364045 5'UTR in PREDICTED: Homo sapiens similar to RAB4B, member

3HSAR050549 CR318270 3'UTR in Homo sapiens FLJ32214 protein (FLJ32214), mRNA.

3HSAR048194 CR308339 3'UTR in Homo sapiens chromosome 19 open reading frame 31

3HSAR045381 CR294946 3'UTR in Homo sapiens hypothetical protein HSPC268

3PTRR006303 CR207805 3'UTR in PREDICTED: Pan troglodytes similar to chromosome 17

3HSAR042414 CR158727 3'UTR in Homo sapiens oxoglutarate (alpha-ketoglutarate)

3HSAR058142 CR379253 3'UTR in Homo sapiens cholinergic receptor, nicotinic, beta

3HSAR062529 CR412983 3'UTR in PREDICTED: Homo sapiens hypothetical protein

3HSAR060101 CR398042 3'UTR in PREDICTED: Homo sapiens hypothetical protein

>3HSAR025295 CR099964 3'UTR in Homo sapiens baculoviral IAP repeat-containing 4

(BIRC4), mRNA.

Length = 6791

Score = 359 bits (181), Expect = 3e-97

Identities = 249/271 (91%), Gaps = 3/271 (1%)

Strand = Plus / Plus

Query: 860 ggcgctgtggctcacgcctgtaatcccagcactttgggaggccgaggcaggcggatcacc 919

||||| |||||||| ||||||||||||||||||||||||||||||||| ||| |||||||

Sbjct: 4020 ggcgcggtggctcatgcctgtaatcccagcactttgggaggccgaggcgggcagatcacc 4079

Query: 920 tgaggtcgggagtccgagaccagcctgaccaacatggagaaaccccgtctctactaaaaa 979

|||||||||||| ||||||||||||||||||||||||||||||||||||||||||||||

Sbjct: 4080 tgaggtcgggaggtcgagaccagcctgaccaacatggagaaaccccgtctctactaaaaa 4139

Query: 980 tacaaaattagctgggcgtggtggcgcatg---gtagtcccagctgctccggaggctgag 1036

||||||||||||||||||||||||| |||| ||| |||||||| || ||||||||||

Sbjct: 4140 tacaaaattagctgggcgtggtggctcatgcctgtaatcccagctacttgggaggctgag 4199

Query: 1037 gcaggagaatcgcttgaacccgggaggcagaggttgcggtgagccgagatcacgccattg 1096

||||||||||||||||||||| |||||| ||||||| ||||||| ||||| |||||||

Sbjct: 4200 gcaggagaatcgcttgaacccaggaggcggaggttgtggtgagcgaagatcgtgccattg 4259

Query: 1097 cactccaacctgggcaacaagagcaaaactc 1127

||||||| |||||||||||||||||||||||

Sbjct: 4260 cactccagcctgggcaacaagagcaaaactc 4290

Score = 232 bits (117), Expect = 5e-59

Identities = 222/254 (87%), Gaps = 6/254 (2%)

Strand = Plus / Plus

Query: 866 gtggctcacgcctgtaatcccagcactttgggaggccgaggcaggcggatcacctgaggt 925

|||||||||||||||||||||||||||||||||||| |||||||| ||||||| |||||

Sbjct: 3418 gtggctcacgcctgtaatcccagcactttgggaggctgaggcaggtggatcac--gaggt 3475

Query: 926 cgggagtccgagaccagcctgaccaacatggagaaaccccgtctctact-aaaaatacaa 984

| |||| |||||||| |||| | |||| || ||||||||||||||||| ||||| | ||

Sbjct: 3476 caggagatcgagaccatcctggctaacacggtgaaaccccgtctctactaaaaaacagaa 3535

Query: 985 aattagctgggcgtggtggcgcatg---gtagtcccagctgctccggaggctgaggcagg 1041

||||||| ||||||||||||| | |||||||||||| ||| |||||||||||||||

Sbjct: 3536 aattagccgggcgtggtggcgggcgcctgtagtcccagctactcgggaggctgaggcagg 3595

Query: 1042 agaatcgcttgaacccgggaggcagaggttgcggtgagccgagatcacgccattgcactc 1101

||||| | |||||||||||||||||| |||| ||||||||||||| ||||| |||||||

Sbjct: 3596 agaatggtgtgaacccgggaggcagagcttgcagtgagccgagatctcgccactgcactc 3655

Query: 1102 caacctgggcaaca 1115

|| |||||||||||

Sbjct: 3656 cagcctgggcaaca 3669

Score = 188 bits (95), Expect = 6e-46

Identities = 206/241 (85%), Gaps = 4/241 (1%)

Strand = Plus / Plus

Query: 866 gtggctcacgcctgtaatcccagcactttgggaggccgaggcaggcggatcacctgaggt 925

||||||||||||||||||||||||| ||| ||||| ||||||||||| |||||||||||

Sbjct: 6020 gtggctcacgcctgtaatcccagcagtttccgaggctgaggcaggcggctcacctgaggt 6079

Query: 926 cgggagtccgagaccagcctgaccaacatggagaaaccccgtctctactaaaaatacaaa 985

| ||||| |||||||||||| |||||||| ||||||| |||| ||| |||||||||||

Sbjct: 6080 caggagttggagaccagcctggccaacatgctgaaaccctgtctgtac-aaaaatacaaa 6138

Query: 986 attagctgggcgtggtggcgcatg---gtagtcccagctgctccggaggctgaggcagga 1042

| ||||||||| |||||||||||| |||||||||||| || | | |||||||||||

Sbjct: 6139 aatagctgggcatggtggcgcatgcctgtagtcccagctactagagcgactgaggcagga 6198

Query: 1043 gaatcgcttgaacccgggaggcagaggttgcggtgagccgagatcacgccattgcactcc 1102

|||| ||||||||| ||||||| |||||||| | ||||| |||| ||||| |||||||

Sbjct: 6199 gaattgcttgaacctgggaggcggaggttgcagggagccaagatggcgccaccgcactcc 6258

Query: 1103 a 1103

|

Sbjct: 6259 a 6259

>5HSAR057161 BR364045 5'UTR in PREDICTED: Homo sapiens similar to RAB4B, member

RAS oncogene family (LOC654335), mRNA.

Length = 856

Score = 349 bits (176), Expect = 3e-94

Identities = 238/258 (92%), Gaps = 3/258 (1%)

Strand = Plus / Plus

Query: 860 ggcgctgtggctcacgcctgtaatcccagcactttgggaggccgaggcaggcggatcacc 919

||||| |||||||| ||||||||||||||||||||||||||| |||||||||||||||||

Sbjct: 426 ggcgcggtggctcatgcctgtaatcccagcactttgggaggctgaggcaggcggatcacc 485

Query: 920 tgaggtcgggagtccgagaccagcctgaccaacatggagaaaccccgtctctactaaaaa 979

||||||||||||| |||||||||||||||||||||||||||||||||||||||||||||

Sbjct: 486 tgaggtcgggagtttgagaccagcctgaccaacatggagaaaccccgtctctactaaaaa 545

Query: 980 tacaaaattagctgggcgtggtggcgcatg---gtagtcccagctgctccggaggctgag 1036

|||| ||||||||| | ||||||||||||| ||| |||||||| ||| ||||||||||

Sbjct: 546 tacagaattagctgagtgtggtggcgcatgcctgtaatcccagctactcgggaggctgag 605

Query: 1037 gcaggagaatcgcttgaacccgggaggcagaggttgcggtgagccgagatcacgccattg 1096

|||||||||| ||||||||| |||||| |||||||||||||||||||||| ||||||||

Sbjct: 606 gcaggagaattgcttgaacctgggaggtggaggttgcggtgagccgagatcgcgccattg 665

Query: 1097 cactccaacctgggcaac 1114

||||||| ||||||||||

Sbjct: 666 cactccagcctgggcaac 683

>3HSAR050549 CR318270 3'UTR in Homo sapiens FLJ32214 protein (FLJ32214), mRNA.

Length = 1055

Score = 347 bits (175), Expect = 1e-93

Identities = 237/257 (92%), Gaps = 3/257 (1%)

Strand = Plus / Plus

Query: 866 gtggctcacgcctgtaatcccagcactttgggaggccgaggcaggcggatcacctgaggt 925

|||||||||||||||||||||| ||||||||||||| ||||||||| |||||||||||||

Sbjct: 785 gtggctcacgcctgtaatcccaacactttgggaggctgaggcaggcagatcacctgaggt 844

Query: 926 cgggagtccgagaccagcctgaccaacatggagaaaccccgtctctactaaaaatacaaa 985

|||||| ||||||||||||||||||||||||||||||||||||||||||||||||||||

Sbjct: 845 cgggagcgcgagaccagcctgaccaacatggagaaaccccgtctctactaaaaatacaaa 904

Query: 986 attagctgggcgtggtggcgcatg---gtagtcccagctgctccggaggctgaggcagga 1042

|||||||||||||||||||||||| ||| |||||||| || ||||||||||||||||

Sbjct: 905 attagctgggcgtggtggcgcatgcctgtaatcccagctacttgggaggctgaggcagga 964

Query: 1043 gaatcgcttgaacccgggaggcagaggttgcggtgagccgagatcacgccattgcactcc 1102

|||||||||||||||||||||||||||||| | |||||| ||||| |||||| ||||||

Sbjct: 965 gaatcgcttgaacccgggaggcagaggttgtgttgagccaagatcgtgccattccactcc 1024

Query: 1103 aacctgggcaacaagag 1119

| |||||||||| ||||

Sbjct: 1025 agcctgggcaacgagag 1041

>3HSAR048194 CR308339 3'UTR in Homo sapiens chromosome 19 open reading frame 31

(C19orf31), mRNA.

Length = 1474

Score = 345 bits (174), Expect = 4e-93

Identities = 242/264 (91%), Gaps = 3/264 (1%)

Strand = Plus / Plus

Query: 867 tggctcacgcctgtaatcccagcactttgggaggccgaggcaggcggatcacctgaggtc 926

|||||||| |||||||||||||||||||||||||||||||||||| ||||||||||||||

Sbjct: 1205 tggctcacacctgtaatcccagcactttgggaggccgaggcaggcagatcacctgaggtc 1264

Query: 927 gggagtccgagaccagcctgaccaacatggagaaaccccgtctctactaaaaatacaaaa 986

||||| ||||||||||||||| |||||||||||||||||| |||||||| || ||||||

Sbjct: 1265 aggagttcgagaccagcctgactaacatggagaaaccccgtttctactaagaacacaaaa 1324

Query: 987 ttagctgggcgtggtggcgcatg---gtagtcccagctgctccggaggctgaggcaggag 1043

||||| ||||||||||| |||| ||| |||||||| ||| |||||||||||||||||

Sbjct: 1325 ttagcctggcgtggtggcacatgtctgtaatcccagctactcgggaggctgaggcaggag 1384

Query: 1044 aatcgcttgaacccgggaggcagaggttgcggtgagccgagatcacgccattgcactcca 1103

||||||||||||||||||||||||||||| |||||||| ||||| |||||||||||||||

Sbjct: 1385 aatcgcttgaacccgggaggcagaggttgtggtgagccaagatcgcgccattgcactcca 1444

Query: 1104 acctgggcaacaagagcaaaactc 1127

|||||||||||| ||||||||||

Sbjct: 1445 gcctgggcaacaaaagcaaaactc 1468

>3HSAR045381 CR294946 3'UTR in Homo sapiens hypothetical protein HSPC268

(HSPC268), mRNA.

Length = 646

Score = 341 bits (172), Expect = 7e-92

Identities = 253/279 (90%), Gaps = 7/279 (2%)

Strand = Plus / Plus

Query: 856 gccaggcgctgtggctcacgcctgtaatcccagcactttgggaggccgaggcaggcggat 915

||||||| | ||||||||||||||||||||||||||||||||||||| ||||||||||||

Sbjct: 253 gccaggcacggtggctcacgcctgtaatcccagcactttgggaggccaaggcaggcggat 312

Query: 916 cacctgaggtcgggagtccgagaccagcctgaccaacatggagaaaccccgtctctacta 975

||||||||||||||||| || ||||||||||||||||||||| |||||||||||||||||

Sbjct: 313 cacctgaggtcgggagttcgcgaccagcctgaccaacatggaaaaaccccgtctctacta 372

Query: 976 aaaatacaaaattagctgggcgtggtggcgcatg---gtagtcccagctgctccggaggc 1032

|||||||||||||||| ||||||||||| |||| |||||||||||| ||| ||||||

Sbjct: 373 aaaatacaaaattagccaggcgtggtggcacatgcctgtagtcccagctactcaggaggc 432

Query: 1033 tgaggcaggagaatcgcttgaacccgggaggcagaggttgcggtgagccgagatcacgcc 1092

|| | |||||||||||||||||||||||||| ||||||||||||||| |||||| ||||

Sbjct: 433 tgggacaggagaatcgcttgaacccgggaggtggaggttgcggtgagctgagatcgcgcc 492

Query: 1093 attg----cactccaacctgggcaacaagagcaaaactc 1127

|| | ||||||| |||||||||||||||||||||||

Sbjct: 493 atggcactcactccagcctgggcaacaagagcaaaactc 531

>3PTRR006303 CR207805 3'UTR in PREDICTED: Pan troglodytes similar to chromosome 17

open reading frame 27 (LOC454944), mRNA.

Length = 345

Score = 339 bits (171), Expect = 3e-91

Identities = 251/277 (90%), Gaps = 3/277 (1%)

Strand = Plus / Plus

Query: 855 ggccaggcgctgtggctcacgcctgtaatcccagcactttgggaggccgaggcaggcgga 914

|||| ||||| |||||||||||||||||||||||||||||||||||| ||||| || | |

Sbjct: 48 ggccgggcgcggtggctcacgcctgtaatcccagcactttgggaggctgaggcgggggaa 107

Query: 915 tcacctgaggtcgggagtccgagaccagcctgaccaacatggagaaaccccgtctctact 974

|||||||||||||||||| ||||||||||||||||||||||||||||||| |||||||||

Sbjct: 108 tcacctgaggtcgggagttcgagaccagcctgaccaacatggagaaacccggtctctact 167

Query: 975 aaaaatacaaaattagctgggcgtggtggcgcatg---gtagtcccagctgctccggagg 1031

|||||||||||||||| ||| ||||||| ||||| ||| |||||||| ||| | |||

Sbjct: 168 aaaaatacaaaattagacgggtgtggtggtgcatgcctgtaatcccagctactcggaagg 227

Query: 1032 ctgaggcaggagaatcgcttgaacccgggaggcagaggttgcggtgagccgagatcacgc 1091

||||||| ||||||||||||||||||||||||| |||||||||||||||| | |||||||

Sbjct: 228 ctgaggcgggagaatcgcttgaacccgggaggcggaggttgcggtgagccaacatcacgc 287

Query: 1092 cattgcactccaacctgggcaacaagagcaaaactcc 1128

|||||||||||| ||| |||||||||||| |||||||

Sbjct: 288 cattgcactccagcctcggcaacaagagcgaaactcc 324

>3HSAR042414 CR158727 3'UTR in Homo sapiens oxoglutarate (alpha-ketoglutarate)

dehydrogenase (lipoamide) (OGDH), nuclear gene encoding

mitochondrial protein, transcript variant 2, mRNA.

Length = 398

Score = 333 bits (168), Expect = 2e-89

Identities = 252/278 (90%), Gaps = 4/278 (1%)

Strand = Plus / Plus

Query: 854 cggccaggcgctgtggctcacgcctgtaatcccagcactttgggaggccgaggcaggcgg 913

||||| ||||| ||| ||||||||||||||||||||||||||||||||||||| ||| |

Sbjct: 46 cggccgggcgcagtgtctcacgcctgtaatcccagcactttgggaggccgaggtgggcag 105

Query: 914 atcacctgaggtcgggagtccgagaccagcctgaccaacatggagaaaccccgtctctac 973

|||||||||| |||||||| |||||||||||||||||||||||||||| || |||||||

Sbjct: 106 atcacctgagttcgggagttggagaccagcctgaccaacatggagaaactccatctctac 165

Query: 974 taaaaatacaaaattagctgggcgtggtggcgcatg---gtagtcccagctgctccggag 1030

||||||||||||||||||||||||||||||||| | ||| |||||||| ||| ||||

Sbjct: 166 taaaaatacaaaattagctgggcgtggtggcgcgcgcctgtaatcccagctactcaggag 225

Query: 1031 gctgaggcaggagaatcgcttgaacccgggaggcagaggttgcggtgagccgagatcacg 1090

||||||||||||||||||||||||||||||||| |||||||| |||||||||||| |||

Sbjct: 226 gctgaggcaggagaatcgcttgaacccgggaggtggaggttgcagtgagccgagattacg 285

Query: 1091 ccattgcactccaacctggg-caacaagagcaaaactc 1127

||||||||||||| |||||| |||||||||| ||||||

Sbjct: 286 ccattgcactccagcctgggccaacaagagcgaaactc 323

>3HSAR058142 CR379253 3'UTR in Homo sapiens cholinergic receptor, nicotinic, beta

1 (muscle) (CHRNB1), mRNA.

Length = 890

Score = 331 bits (167), Expect = 7e-89

Identities = 250/277 (90%), Gaps = 3/277 (1%)

Strand = Plus / Plus

Query: 855 ggccaggcgctgtggctcacgcctgtaatcccagcactttgggaggccgaggcaggcgga 914

|||| ||||| ||||||||||||||||||||||||||||||||||||| |||| ||||||

Sbjct: 313 ggccgggcgcggtggctcacgcctgtaatcccagcactttgggaggccaaggcgggcgga 372

Query: 915 tcacctgaggtcgggagtccgagaccagcctgaccaacatggagaaaccccgtctctact 974

|||||||||||||||||| |||||||||| ||||||||||||||||||| |||||||||

Sbjct: 373 tcacctgaggtcgggagtttgagaccagcccgaccaacatggagaaaccctgtctctact 432

Query: 975 aaaaatacaaaattagctgggcgtggtggcgcatg---gtagtcccagctgctccggagg 1031

||||||||||||||||| || ||||||| |||| ||| |||||||| || |||||

Sbjct: 433 aaaaatacaaaattagccaggtgtggtggtacatgcctgtaatcccagctactagggagg 492

Query: 1032 ctgaggcaggagaatcgcttgaacccgggaggcagaggttgcggtgagccgagatcacgc 1091

|||||||||||||||| || |||||||||||||||||||||| ||||| | |||||||||

Sbjct: 493 ctgaggcaggagaatcactcgaacccgggaggcagaggttgcagtgagtcaagatcacgc 552

Query: 1092 cattgcactccaacctgggcaacaagagcaaaactcc 1128

|||||||||||| |||||||||||||||| |||||||

Sbjct: 553 cattgcactccagcctgggcaacaagagcgaaactcc 589

>3HSAR062529 CR412983 3'UTR in PREDICTED: Homo sapiens hypothetical protein

FLJ37228 (FLJ37228), mRNA.

Length = 2009

Score = 329 bits (166), Expect = 3e-88

Identities = 252/280 (90%), Gaps = 3/280 (1%)

Strand = Plus / Plus

Query: 855 ggccaggcgctgtggctcacgcctgtaatcccagcactttgggaggccgaggcaggcgga 914

|||| ||||| |||||||| ||||||||||||||||||||||||||||||||| || |||

Sbjct: 350 ggccgggcgcagtggctcatgcctgtaatcccagcactttgggaggccgaggcgggtgga 409

Query: 915 tcacctgaggtcgggagtccgagaccagcctgaccaacatggagaaaccccgtctctact 974

|||||||||| | ||||| | |||||||||||||||||||||||||||||| ||||||||

Sbjct: 410 tcacctgaggccaggagttcaagaccagcctgaccaacatggagaaaccccatctctact 469

Query: 975 aaaaatacaaaattagctgggcgtggtggcgcatg---gtagtcccagctgctccggagg 1031

||||||||||||||||| || |||||| ||||| ||| |||||||| | |||||

Sbjct: 470 aaaaatacaaaattagccaggtgtggtgttgcatgcctgtaatcccagctattagggagg 529

Query: 1032 ctgaggcaggagaatcgcttgaacccgggaggcagaggttgcggtgagccgagatcacgc 1091

|||||||||||||||||||||||||||||||||||||||||| ||||| ||||||||| |

Sbjct: 530 ctgaggcaggagaatcgcttgaacccgggaggcagaggttgcagtgagtcgagatcacac 589

Query: 1092 cattgcactccaacctgggcaacaagagcaaaactccgtc 1131

|||||||||||| ||||||||| |||||||||||||||||

Sbjct: 590 cattgcactccagcctgggcaataagagcaaaactccgtc 629

>3HSAR060101 CR398042 3'UTR in PREDICTED: Homo sapiens hypothetical protein

FLJ37228 (FLJ37228), mRNA.

Length = 2009

Score = 329 bits (166), Expect = 3e-88

Identities = 252/280 (90%), Gaps = 3/280 (1%)

Strand = Plus / Plus

Query: 855 ggccaggcgctgtggctcacgcctgtaatcccagcactttgggaggccgaggcaggcgga 914

|||| ||||| |||||||| ||||||||||||||||||||||||||||||||| || |||

Sbjct: 350 ggccgggcgcagtggctcatgcctgtaatcccagcactttgggaggccgaggcgggtgga 409

Query: 915 tcacctgaggtcgggagtccgagaccagcctgaccaacatggagaaaccccgtctctact 974

|||||||||| | ||||| | |||||||||||||||||||||||||||||| ||||||||

Sbjct: 410 tcacctgaggccaggagttcaagaccagcctgaccaacatggagaaaccccatctctact 469

Query: 975 aaaaatacaaaattagctgggcgtggtggcgcatg---gtagtcccagctgctccggagg 1031

||||||||||||||||| || |||||| ||||| ||| |||||||| | |||||

Sbjct: 470 aaaaatacaaaattagccaggtgtggtgttgcatgcctgtaatcccagctattagggagg 529

Query: 1032 ctgaggcaggagaatcgcttgaacccgggaggcagaggttgcggtgagccgagatcacgc 1091

|||||||||||||||||||||||||||||||||||||||||| ||||| ||||||||| |

Sbjct: 530 ctgaggcaggagaatcgcttgaacccgggaggcagaggttgcagtgagtcgagatcacac 589

Query: 1092 cattgcactccaacctgggcaacaagagcaaaactccgtc 1131

|||||||||||| ||||||||| |||||||||||||||||

Sbjct: 590 cattgcactccagcctgggcaataagagcaaaactccgtc 629

Query= ncINT55as

Database: UTRef all

463,416 sequences; 192,534,142 total letters

3HSAR042316 CR158421 3'UTR in Homo sapiens multimerin 2 (MMRN2), mRNA.

3HSAR039567 CR150907 3'UTR in Homo sapiens methylthioadenosine phosphorylase

3HSAR061792 CR409385 3'UTR in PREDICTED: Homo sapiens hypothetical gene supported

3HSAR060453 CR400651 3'UTR in PREDICTED: Homo sapiens hypothetical gene supported

3HSAR065254 CR428862 3'UTR in PREDICTED: Homo sapiens similar to Ribosome

3HSAR065135 CR428443 3'UTR in PREDICTED: Homo sapiens similar to Ribosome

3HSAR055471 CR358350 3'UTR in PREDICTED: Homo sapiens similar to Ribosome

5HSAR061662 BR392738 5'UTR in PREDICTED: Homo sapiens similar to centaurin,

5HSAR061536 BR392213 5'UTR in PREDICTED: Homo sapiens similar to centaurin,

5HSAR061504 BR391994 5'UTR in PREDICTED: Homo sapiens similar to centaurin,

5HSAR061465 BR391752 5'UTR in PREDICTED: Homo sapiens similar to centaurin,

5HSAR061288 BR390631 5'UTR in PREDICTED: Homo sapiens similar to Ribosome

5HSAR061144 BR389767 5'UTR in PREDICTED: Homo sapiens similar to centaurin,

5HSAR054532 BR339688 5'UTR in PREDICTED: Homo sapiens similar to centaurin,

3HSAR063000 CR415804 3'UTR in Homo sapiens DEAD (Asp-Glu-Ala-Asp) box polypeptide

5HSAR062671 BR396289 5'UTR in PREDICTED: Homo sapiens hypothetical gene supported

5HSAR062482 BR395703 5'UTR in PREDICTED: Homo sapiens hypothetical gene supported

5HSAR062439 BR395562 5'UTR in PREDICTED: Homo sapiens hypothetical gene supported

5HSAR062397 BR395423 5'UTR in PREDICTED: Homo sapiens hypothetical gene supported

5HSAR062295 BR395071 5'UTR in PREDICTED: Homo sapiens hypothetical gene supported

3HSAR064445 CR425039 3'UTR in PREDICTED: Homo sapiens hypothetical protein

3HSAR053129 CR344969 3'UTR in PREDICTED: Homo sapiens hypothetical protein

5HSAR061981 BR393975 5'UTR in PREDICTED: Homo sapiens similar to ARF

5HSAR061518 BR392135 5'UTR in PREDICTED: Homo sapiens similar to ARF

3HSAR005497 CR039782 3'UTR in Homo sapiens sialic acid binding Ig-like lectin 8

5HSAR061927 BR393793 5'UTR in PREDICTED: Homo sapiens similar to ARF

5HSAR061688 BR392848 5'UTR in PREDICTED: Homo sapiens similar to ARF

5HSAR061644 BR392668 5'UTR in PREDICTED: Homo sapiens similar to ARF

5HSAR061558 BR392315 5'UTR in PREDICTED: Homo sapiens similar to ARF

5HSAR061483 BR391904 5'UTR in PREDICTED: Homo sapiens similar to ARF

5HSAR062721 BR396457 5'UTR in PREDICTED: Homo sapiens hypothetical gene supported

3HSAR062576 CR413216 3'UTR in PREDICTED: Homo sapiens hypothetical protein

3HSAR052637 CR342155 3'UTR in PREDICTED: Homo sapiens hypothetical protein

5HSAR057298 BR365291 5'UTR in PREDICTED: Homo sapiens hypothetical protein

5PTRR004722 BR196016 5'UTR in PREDICTED: Pan troglodytes similar to ADP-dependent

>3HSAR042316 CR158421 3'UTR in Homo sapiens multimerin 2 (MMRN2), mRNA.

Length = 904

Score = 149 bits (75), Expect = 6e-34

Identities = 117/130 (90%), Gaps = 2/130 (1%)

Strand = Plus / Plus

Query: 2604 ttctccttcctgcggccttgtgaagaagatgcttgcttccccttcaccttctgccatgat 2663

||||||||||||| ||||||||||| | |||| |||| |||||| |||||||||||

Sbjct: 777 ttctccttcctgccaccttgtgaagatgc--cttggttcctcttcactgtctgccatgat 834

Query: 2664 tgtaagtttcctgaggcctacccagccatgtggaactgtgagtcaattaaacctctttcc 2723

||||||||||||||||||| |||||||||||||||| |||||||||||||||||||||||

Sbjct: 835 tgtaagtttcctgaggcctccccagccatgtggaacagtgagtcaattaaacctctttcc 894

Query: 2724 tttagaaatt 2733

|||| |||||

Sbjct: 895 tttataaatt 904

>3HSAR039567 CR150907 3'UTR in Homo sapiens methylthioadenosine phosphorylase

(MTAP), mRNA.

Length = 3972

Score = 127 bits (64), Expect = 2e-27

Identities = 79/84 (94%)

Strand = Plus / Plus

Query: 2650 ccttctgccatgattgtaagtttcctgaggcctacccagccatgtggaactgtgagtcaa 2709

|||||||||| |||||||||||||||||||||| |||||| |||||||||||||||| ||

Sbjct: 3296 ccttctgccacgattgtaagtttcctgaggccttcccagctatgtggaactgtgagttaa 3355

Query: 2710 ttaaacctctttcctttagaaatt 2733

|||||||||||||||||| |||||

Sbjct: 3356 ttaaacctctttcctttataaatt 3379

>3HSAR061792 CR409385 3'UTR in PREDICTED: Homo sapiens hypothetical gene supported

by BC013370; BC034583, transcript variant 3 (LOC400655),

mRNA.

Length = 3117

Score = 115 bits (58), Expect = 9e-24

Identities = 73/78 (93%)

Strand = Plus / Plus

Query: 2650 ccttctgccatgattgtaagtttcctgaggcctacccagccatgtggaactgtgagtcaa 2709

||||||| ||||||||||||||||||||||||| |||| ||||||| |||||||||||||

Sbjct: 627 ccttctgacatgattgtaagtttcctgaggcctccccaaccatgtgaaactgtgagtcaa 686

Query: 2710 ttaaacctctttccttta 2727

|||||||||||| |||||

Sbjct: 687 ttaaacctcttttcttta 704

Score = 61.9 bits (31), Expect = 1e-07

Identities = 46/51 (90%)

Strand = Plus / Plus

Query: 2506 ggaggtaatggaatcataagggcagtttctcccatgctgttctcatgatag 2556

||||||||| |||||| | ||||||| ||||||| ||||||||||||||||

Sbjct: 487 ggaggtaattgaatcacaggggcagtgtctcccacgctgttctcatgatag 537

>3HSAR060453 CR400651 3'UTR in PREDICTED: Homo sapiens hypothetical gene supported

by BC013370; BC034583, transcript variant 2 (LOC400655),

mRNA.

Length = 3117

Score = 115 bits (58), Expect = 9e-24

Identities = 73/78 (93%)

Strand = Plus / Plus

Query: 2650 ccttctgccatgattgtaagtttcctgaggcctacccagccatgtggaactgtgagtcaa 2709

||||||| ||||||||||||||||||||||||| |||| ||||||| |||||||||||||

Sbjct: 627 ccttctgacatgattgtaagtttcctgaggcctccccaaccatgtgaaactgtgagtcaa 686

Query: 2710 ttaaacctctttccttta 2727

|||||||||||| |||||

Sbjct: 687 ttaaacctcttttcttta 704

Score = 61.9 bits (31), Expect = 1e-07

Identities = 46/51 (90%)

Strand = Plus / Plus

Query: 2506 ggaggtaatggaatcataagggcagtttctcccatgctgttctcatgatag 2556

||||||||| |||||| | ||||||| ||||||| ||||||||||||||||

Sbjct: 487 ggaggtaattgaatcacaggggcagtgtctcccacgctgttctcatgatag 537

>3HSAR065254 CR428862 3'UTR in PREDICTED: Homo sapiens similar to Ribosome

biogenesis protein BMS1 homolog, transcript variant 7

(LOC653471), mRNA.

Length = 4506

Score = 113 bits (57), Expect = 3e-23

Identities = 78/84 (92%), Gaps = 2/84 (2%)

Strand = Plus / Plus

Query: 2650 ccttctgccatgattgtaagtttcctgaggcctacccagccatgtggaactgtgagtcaa 2709

||||||||||||||||||||||||||||||||| |||||||||||| ||| ||||||||

Sbjct: 857 ccttctgccatgattgtaagtttcctgaggcctccccagccatgtgaaac--tgagtcaa 914

Query: 2710 ttaaacctctttcctttagaaatt 2733

||||||||||| |||||| |||||

Sbjct: 915 ttaaacctcttccctttaaaaatt 938

>3HSAR065135 CR428443 3'UTR in PREDICTED: Homo sapiens similar to Ribosome

biogenesis protein BMS1 homolog, transcript variant 5

(LOC653471), mRNA.

Length = 2738

Score = 113 bits (57), Expect = 3e-23

Identities = 78/84 (92%), Gaps = 2/84 (2%)

Strand = Plus / Plus

Query: 2650 ccttctgccatgattgtaagtttcctgaggcctacccagccatgtggaactgtgagtcaa 2709

||||||||||||||||||||||||||||||||| |||||||||||| ||| ||||||||

Sbjct: 857 ccttctgccatgattgtaagtttcctgaggcctccccagccatgtgaaac--tgagtcaa 914

Query: 2710 ttaaacctctttcctttagaaatt 2733

||||||||||| |||||| |||||

Sbjct: 915 ttaaacctcttccctttaaaaatt 938

>3HSAR055471 CR358350 3'UTR in PREDICTED: Homo sapiens similar to Ribosome

biogenesis protein BMS1 homolog, transcript variant 4

(LOC654000), mRNA.

Length = 4504

Score = 113 bits (57), Expect = 3e-23

Identities = 78/84 (92%), Gaps = 2/84 (2%)

Strand = Plus / Plus

Query: 2650 ccttctgccatgattgtaagtttcctgaggcctacccagccatgtggaactgtgagtcaa 2709

||||||||||||||||||||||||||||||||| |||||||||||| ||| ||||||||

Sbjct: 857 ccttctgccatgattgtaagtttcctgaggcctccccagccatgtgaaac--tgagtcaa 914

Query: 2710 ttaaacctctttcctttagaaatt 2733

||||||||||| |||||| |||||

Sbjct: 915 ttaaacctcttccctttaaaaatt 938

>5HSAR061662 BR392738 5'UTR in PREDICTED: Homo sapiens similar to centaurin,

gamma-like family, member 1, transcript variant 12

(LOC653468), mRNA.

Length = 2093

Score = 113 bits (57), Expect = 3e-23

Identities = 78/84 (92%), Gaps = 2/84 (2%)

Strand = Plus / Plus

Query: 2650 ccttctgccatgattgtaagtttcctgaggcctacccagccatgtggaactgtgagtcaa 2709

||||||||||||||||||||||||||||||||| |||||||||||| ||| ||||||||

Sbjct: 627 ccttctgccatgattgtaagtttcctgaggcctccccagccatgtgaaac--tgagtcaa 684

Query: 2710 ttaaacctctttcctttagaaatt 2733

||||||||||| |||||| |||||

Sbjct: 685 ttaaacctcttccctttaaaaatt 708

>5HSAR061536 BR392213 5'UTR in PREDICTED: Homo sapiens similar to centaurin,

gamma-like family, member 1, transcript variant 10

(LOC653468), mRNA.

Length = 2093

Score = 113 bits (57), Expect = 3e-23

Identities = 78/84 (92%), Gaps = 2/84 (2%)

Strand = Plus / Plus

Query: 2650 ccttctgccatgattgtaagtttcctgaggcctacccagccatgtggaactgtgagtcaa 2709

||||||||||||||||||||||||||||||||| |||||||||||| ||| ||||||||

Sbjct: 627 ccttctgccatgattgtaagtttcctgaggcctccccagccatgtgaaac--tgagtcaa 684

Query: 2710 ttaaacctctttcctttagaaatt 2733

||||||||||| |||||| |||||

Sbjct: 685 ttaaacctcttccctttaaaaatt 708

>5HSAR061504 BR391994 5'UTR in PREDICTED: Homo sapiens similar to centaurin,

gamma-like family, member 1, transcript variant 9

(LOC653468), mRNA.

Length = 2093

Score = 113 bits (57), Expect = 3e-23

Identities = 78/84 (92%), Gaps = 2/84 (2%)

Strand = Plus / Plus

Query: 2650 ccttctgccatgattgtaagtttcctgaggcctacccagccatgtggaactgtgagtcaa 2709

||||||||||||||||||||||||||||||||| |||||||||||| ||| ||||||||

Sbjct: 627 ccttctgccatgattgtaagtttcctgaggcctccccagccatgtgaaac--tgagtcaa 684

Query: 2710 ttaaacctctttcctttagaaatt 2733

||||||||||| |||||| |||||

Sbjct: 685 ttaaacctcttccctttaaaaatt 708

>5HSAR061465 BR391752 5'UTR in PREDICTED: Homo sapiens similar to centaurin,

gamma-like family, member 1, transcript variant 8

(LOC653468), mRNA.

Length = 2093

Score = 113 bits (57), Expect = 3e-23

Identities = 78/84 (92%), Gaps = 2/84 (2%)

Strand = Plus / Plus

Query: 2650 ccttctgccatgattgtaagtttcctgaggcctacccagccatgtggaactgtgagtcaa 2709

||||||||||||||||||||||||||||||||| |||||||||||| ||| ||||||||

Sbjct: 627 ccttctgccatgattgtaagtttcctgaggcctccccagccatgtgaaac--tgagtcaa 684

Query: 2710 ttaaacctctttcctttagaaatt 2733

||||||||||| |||||| |||||

Sbjct: 685 ttaaacctcttccctttaaaaatt 708

>5HSAR061288 BR390631 5'UTR in PREDICTED: Homo sapiens similar to Ribosome

biogenesis protein BMS1 homolog, transcript variant 2

(LOC653471), mRNA.

Length = 2093

Score = 113 bits (57), Expect = 3e-23

Identities = 78/84 (92%), Gaps = 2/84 (2%)

Strand = Plus / Plus

Query: 2650 ccttctgccatgattgtaagtttcctgaggcctacccagccatgtggaactgtgagtcaa 2709

||||||||||||||||||||||||||||||||| |||||||||||| ||| ||||||||

Sbjct: 627 ccttctgccatgattgtaagtttcctgaggcctccccagccatgtgaaac--tgagtcaa 684

Query: 2710 ttaaacctctttcctttagaaatt 2733

||||||||||| |||||| |||||

Sbjct: 685 ttaaacctcttccctttaaaaatt 708

>5HSAR061144 BR389767 5'UTR in PREDICTED: Homo sapiens similar to centaurin,

gamma-like family, member 1, transcript variant 2

(LOC653468), mRNA.

Length = 2093

Score = 113 bits (57), Expect = 3e-23

Identities = 78/84 (92%), Gaps = 2/84 (2%)

Strand = Plus / Plus

Query: 2650 ccttctgccatgattgtaagtttcctgaggcctacccagccatgtggaactgtgagtcaa 2709

||||||||||||||||||||||||||||||||| |||||||||||| ||| ||||||||

Sbjct: 627 ccttctgccatgattgtaagtttcctgaggcctccccagccatgtgaaac--tgagtcaa 684

Query: 2710 ttaaacctctttcctttagaaatt 2733

||||||||||| |||||| |||||

Sbjct: 685 ttaaacctcttccctttaaaaatt 708

>5HSAR054532 BR339688 5'UTR in PREDICTED: Homo sapiens similar to centaurin,

gamma-like family, member 1 (LOC650155), mRNA.

Length = 2088

Score = 113 bits (57), Expect = 3e-23

Identities = 78/84 (92%), Gaps = 2/84 (2%)

Strand = Plus / Plus

Query: 2650 ccttctgccatgattgtaagtttcctgaggcctacccagccatgtggaactgtgagtcaa 2709

||||||||||||||||||||||||||||||||| |||||||||||| ||| ||||||||

Sbjct: 627 ccttctgccatgattgtaagtttcctgaggcctccccagccatgtgaaac--tgagtcaa 684

Query: 2710 ttaaacctctttcctttagaaatt 2733

||||||||||| |||||| |||||

Sbjct: 685 ttaaacctcttccctttaaaaatt 708

>3HSAR063000 CR415804 3'UTR in Homo sapiens DEAD (Asp-Glu-Ala-Asp) box polypeptide

58 (DDX58), mRNA.

Length = 1823

Score = 111 bits (56), Expect = 1e-22

Identities = 80/88 (90%)

Strand = Plus / Plus

Query: 2645 cttcaccttctgccatgattgtaagtttcctgaggcctacccagccatgtggaactgtga 2704

|||| ||||||||||||||||||||||||||||||||| | |||||||||||||||||||

Sbjct: 1731 cttccccttctgccatgattgtaagtttcctgaggcctcctcagccatgtggaactgtga 1790

Query: 2705 gtcaattaaacctctttcctttagaaat 2732

|| ||||| ||||||| ||||| ||||

Sbjct: 1791 atctattaagcctcttttctttataaat 1818

>5HSAR062671 BR396289 5'UTR in PREDICTED: Homo sapiens hypothetical gene supported

by AK093334; AL833330; BC020871; BC032492, transcript

variant 13 (LOC399753), mRNA.

Length = 2138

Score = 107 bits (54), Expect = 2e-21

Identities = 75/81 (92%), Gaps = 2/81 (2%)

Strand = Plus / Plus

Query: 2653 tctgccatgattgtaagtttcctgaggcctacccagccatgtggaactgtgagtcaatta 2712

|||||||||||||||||||||||||||||| |||||||||||| ||| |||||||||||

Sbjct: 668 tctgccatgattgtaagtttcctgaggcctccccagccatgtgaaac--tgagtcaatta 725

Query: 2713 aacctctttcctttagaaatt 2733

| ||||||||||||| |||||

Sbjct: 726 agcctctttcctttaaaaatt 746

Score = 56.0 bits (28), Expect = 7e-06

Identities = 28/28 (100%)

Strand = Plus / Plus

Query: 2650 ccttctgccatgattgtaagtttcctga 2677

||||||||||||||||||||||||||||

Sbjct: 628 ccttctgccatgattgtaagtttcctga 655

>5HSAR062482 BR395703 5'UTR in PREDICTED: Homo sapiens hypothetical gene supported

by AK093334; AL833330; BC020871; BC032492, transcript

variant 9 (LOC399753), mRNA.

Length = 2138

Score = 107 bits (54), Expect = 2e-21

Identities = 75/81 (92%), Gaps = 2/81 (2%)

Strand = Plus / Plus

Query: 2653 tctgccatgattgtaagtttcctgaggcctacccagccatgtggaactgtgagtcaatta 2712

|||||||||||||||||||||||||||||| |||||||||||| ||| |||||||||||

Sbjct: 668 tctgccatgattgtaagtttcctgaggcctccccagccatgtgaaac--tgagtcaatta 725

Query: 2713 aacctctttcctttagaaatt 2733

| ||||||||||||| |||||

Sbjct: 726 agcctctttcctttaaaaatt 746

Score = 56.0 bits (28), Expect = 7e-06

Identities = 28/28 (100%)

Strand = Plus / Plus

Query: 2650 ccttctgccatgattgtaagtttcctga 2677

||||||||||||||||||||||||||||

Sbjct: 628 ccttctgccatgattgtaagtttcctga 655

>5HSAR062439 BR395562 5'UTR in PREDICTED: Homo sapiens hypothetical gene supported

by AK093334; AL833330; BC020871; BC032492, transcript

variant 8 (LOC399753), mRNA.

Length = 2138

Score = 107 bits (54), Expect = 2e-21

Identities = 75/81 (92%), Gaps = 2/81 (2%)

Strand = Plus / Plus

Query: 2653 tctgccatgattgtaagtttcctgaggcctacccagccatgtggaactgtgagtcaatta 2712

|||||||||||||||||||||||||||||| |||||||||||| ||| |||||||||||

Sbjct: 668 tctgccatgattgtaagtttcctgaggcctccccagccatgtgaaac--tgagtcaatta 725

Query: 2713 aacctctttcctttagaaatt 2733

| ||||||||||||| |||||

Sbjct: 726 agcctctttcctttaaaaatt 746

Score = 56.0 bits (28), Expect = 7e-06

Identities = 28/28 (100%)

Strand = Plus / Plus

Query: 2650 ccttctgccatgattgtaagtttcctga 2677

||||||||||||||||||||||||||||

Sbjct: 628 ccttctgccatgattgtaagtttcctga 655

>5HSAR062397 BR395423 5'UTR in PREDICTED: Homo sapiens hypothetical gene supported

by AK093334; AL833330; BC020871; BC032492, transcript

variant 7 (LOC399753), mRNA.

Length = 2138

Score = 107 bits (54), Expect = 2e-21

Identities = 75/81 (92%), Gaps = 2/81 (2%)

Strand = Plus / Plus

Query: 2653 tctgccatgattgtaagtttcctgaggcctacccagccatgtggaactgtgagtcaatta 2712

|||||||||||||||||||||||||||||| |||||||||||| ||| |||||||||||

Sbjct: 668 tctgccatgattgtaagtttcctgaggcctccccagccatgtgaaac--tgagtcaatta 725

Query: 2713 aacctctttcctttagaaatt 2733

| ||||||||||||| |||||

Sbjct: 726 agcctctttcctttaaaaatt 746

Score = 56.0 bits (28), Expect = 7e-06

Identities = 28/28 (100%)

Strand = Plus / Plus

Query: 2650 ccttctgccatgattgtaagtttcctga 2677

||||||||||||||||||||||||||||

Sbjct: 628 ccttctgccatgattgtaagtttcctga 655

>5HSAR062295 BR395071 5'UTR in PREDICTED: Homo sapiens hypothetical gene supported

by AK093334; AL833330; BC020871; BC032492, transcript

variant 5 (LOC399753), mRNA.

Length = 2138

Score = 107 bits (54), Expect = 2e-21

Identities = 75/81 (92%), Gaps = 2/81 (2%)

Strand = Plus / Plus

Query: 2653 tctgccatgattgtaagtttcctgaggcctacccagccatgtggaactgtgagtcaatta 2712

|||||||||||||||||||||||||||||| |||||||||||| ||| |||||||||||

Sbjct: 668 tctgccatgattgtaagtttcctgaggcctccccagccatgtgaaac--tgagtcaatta 725

Query: 2713 aacctctttcctttagaaatt 2733

| ||||||||||||| |||||

Sbjct: 726 agcctctttcctttaaaaatt 746

Score = 56.0 bits (28), Expect = 7e-06

Identities = 28/28 (100%)

Strand = Plus / Plus

Query: 2650 ccttctgccatgattgtaagtttcctga 2677

||||||||||||||||||||||||||||

Sbjct: 628 ccttctgccatgattgtaagtttcctga 655

>3HSAR064445 CR425039 3'UTR in PREDICTED: Homo sapiens hypothetical protein

LOC652244 (LOC652244), mRNA.

Length = 1505

Score = 105 bits (53), Expect = 8e-21

Identities = 62/65 (95%)

Strand = Plus / Plus

Query: 2657 ccatgattgtaagtttcctgaggcctacccagccatgtggaactgtgagtcaattaaacc 2716

|||||||| |||||||||||||| || |||||||||||||||||||||||||||||||||

Sbjct: 1437 ccatgattttaagtttcctgagggctccccagccatgtggaactgtgagtcaattaaacc 1496

Query: 2717 tcttt 2721

|||||

Sbjct: 1497 tcttt 1501

>3HSAR053129 CR344969 3'UTR in PREDICTED: Homo sapiens hypothetical protein

LOC645293 (LOC645293), mRNA.

Length = 1505

Score = 105 bits (53), Expect = 8e-21

Identities = 62/65 (95%)

Strand = Plus / Plus

Query: 2657 ccatgattgtaagtttcctgaggcctacccagccatgtggaactgtgagtcaattaaacc 2716

|||||||| |||||||||||||| || |||||||||||||||||||||||||||||||||

Sbjct: 1437 ccatgattttaagtttcctgagggctccccagccatgtggaactgtgagtcaattaaacc 1496

Query: 2717 tcttt 2721

|||||

Sbjct: 1497 tcttt 1501

>5HSAR061981 BR393975 5'UTR in PREDICTED: Homo sapiens similar to ARF

GTPase-activating protein, transcript variant 14

(FLJ00312), mRNA.

Length = 2094

Score = 105 bits (53), Expect = 8e-21

Identities = 78/84 (92%), Gaps = 3/84 (3%)

Strand = Plus / Plus

Query: 2650 ccttctgccatgattgtaagtttcctgaggcctacccagccatgtggaactgtgagtcaa 2709

||||||||||||||||||| ||||||||||||| |||||||||||| ||||| ||||||

Sbjct: 623 ccttctgccatgattgtaa-tttcctgaggcctccccagccatgtgaaactg--agtcaa 679

Query: 2710 ttaaacctctttcctttagaaatt 2733

|||||||||||||||||| |||||

Sbjct: 680 ttaaacctctttcctttaaaaatt 703

>5HSAR061518 BR392135 5'UTR in PREDICTED: Homo sapiens similar to ARF

GTPase-activating protein, transcript variant 6

(FLJ00312), mRNA.

Length = 2094

Score = 105 bits (53), Expect = 8e-21

Identities = 78/84 (92%), Gaps = 3/84 (3%)

Strand = Plus / Plus

Query: 2650 ccttctgccatgattgtaagtttcctgaggcctacccagccatgtggaactgtgagtcaa 2709

||||||||||||||||||| ||||||||||||| |||||||||||| ||||| ||||||

Sbjct: 623 ccttctgccatgattgtaa-tttcctgaggcctccccagccatgtgaaactg--agtcaa 679

Query: 2710 ttaaacctctttcctttagaaatt 2733

|||||||||||||||||| |||||

Sbjct: 680 ttaaacctctttcctttaaaaatt 703

>3HSAR005497 CR039782 3'UTR in Homo sapiens sialic acid binding Ig-like lectin 8

(SIGLEC8), mRNA.

Length = 1544

Score = 101 bits (51), Expect = 1e-19

Identities = 60/63 (95%)

Strand = Plus / Plus

Query: 2650 ccttctgccatgattgtaagtttcctgaggcctacccagccatgtggaactgtgagtcaa 2709

||||| ||||||||||||||||||||||||||| ||| ||||||||||||||||||||||

Sbjct: 1442 ccttccgccatgattgtaagtttcctgaggcctcccccgccatgtggaactgtgagtcaa 1501

Query: 2710 tta 2712

|||

Sbjct: 1502 tta 1504

>5HSAR061927 BR393793 5'UTR in PREDICTED: Homo sapiens similar to ARF

GTPase-activating protein, transcript variant 13

(FLJ00312), mRNA.

Length = 2133

Score = 101 bits (51), Expect = 1e-19

Identities = 76/82 (92%), Gaps = 3/82 (3%)

Strand = Plus / Plus

Query: 2653 tctgccatgattgt-aagtttcctgaggcctacccagccatgtggaactgtgagtcaatt 2711

|||||||||||||| |||||||||||||||| |||||||||||| ||| ||||||||||

Sbjct: 663 tctgccatgattgttaagtttcctgaggcctccccagccatgtgaaac--tgagtcaatt 720

Query: 2712 aaacctctttcctttagaaatt 2733

|||||||||||||||| |||||

Sbjct: 721 aaacctctttcctttaaaaatt 742

Score = 56.0 bits (28), Expect = 7e-06

Identities = 28/28 (100%)

Strand = Plus / Plus

Query: 2650 ccttctgccatgattgtaagtttcctga 2677

||||||||||||||||||||||||||||

Sbjct: 623 ccttctgccatgattgtaagtttcctga 650

>5HSAR061688 BR392848 5'UTR in PREDICTED: Homo sapiens similar to ARF

GTPase-activating protein, transcript variant 9

(FLJ00312), mRNA.

Length = 2133

Score = 101 bits (51), Expect = 1e-19

Identities = 76/82 (92%), Gaps = 3/82 (3%)

Strand = Plus / Plus

Query: 2653 tctgccatgattgt-aagtttcctgaggcctacccagccatgtggaactgtgagtcaatt 2711

|||||||||||||| |||||||||||||||| |||||||||||| ||| ||||||||||

Sbjct: 663 tctgccatgattgttaagtttcctgaggcctccccagccatgtgaaac--tgagtcaatt 720

Query: 2712 aaacctctttcctttagaaatt 2733

|||||||||||||||| |||||

Sbjct: 721 aaacctctttcctttaaaaatt 742

Score = 56.0 bits (28), Expect = 7e-06

Identities = 28/28 (100%)

Strand = Plus / Plus

Query: 2650 ccttctgccatgattgtaagtttcctga 2677

||||||||||||||||||||||||||||

Sbjct: 623 ccttctgccatgattgtaagtttcctga 650

>5HSAR061644 BR392668 5'UTR in PREDICTED: Homo sapiens similar to ARF

GTPase-activating protein, transcript variant 2

(FLJ00312), mRNA.

Length = 2133

Score = 101 bits (51), Expect = 1e-19

Identities = 76/82 (92%), Gaps = 3/82 (3%)

Strand = Plus / Plus

Query: 2653 tctgccatgattgt-aagtttcctgaggcctacccagccatgtggaactgtgagtcaatt 2711

|||||||||||||| |||||||||||||||| |||||||||||| ||| ||||||||||

Sbjct: 663 tctgccatgattgttaagtttcctgaggcctccccagccatgtgaaac--tgagtcaatt 720

Query: 2712 aaacctctttcctttagaaatt 2733

|||||||||||||||| |||||

Sbjct: 721 aaacctctttcctttaaaaatt 742

Score = 56.0 bits (28), Expect = 7e-06

Identities = 28/28 (100%)

Strand = Plus / Plus

Query: 2650 ccttctgccatgattgtaagtttcctga 2677

||||||||||||||||||||||||||||

Sbjct: 623 ccttctgccatgattgtaagtttcctga 650

>5HSAR061558 BR392315 5'UTR in PREDICTED: Homo sapiens similar to ARF

GTPase-activating protein, transcript variant 7

(FLJ00312), mRNA.

Length = 2133

Score = 101 bits (51), Expect = 1e-19

Identities = 76/82 (92%), Gaps = 3/82 (3%)

Strand = Plus / Plus

Query: 2653 tctgccatgattgt-aagtttcctgaggcctacccagccatgtggaactgtgagtcaatt 2711

|||||||||||||| |||||||||||||||| |||||||||||| ||| ||||||||||

Sbjct: 663 tctgccatgattgttaagtttcctgaggcctccccagccatgtgaaac--tgagtcaatt 720

Query: 2712 aaacctctttcctttagaaatt 2733

|||||||||||||||| |||||

Sbjct: 721 aaacctctttcctttaaaaatt 742

Score = 56.0 bits (28), Expect = 7e-06

Identities = 28/28 (100%)

Strand = Plus / Plus

Query: 2650 ccttctgccatgattgtaagtttcctga 2677

||||||||||||||||||||||||||||

Sbjct: 623 ccttctgccatgattgtaagtttcctga 650

>5HSAR061483 BR391904 5'UTR in PREDICTED: Homo sapiens similar to ARF

GTPase-activating protein, transcript variant 5

(FLJ00312), mRNA.

Length = 2133

Score = 101 bits (51), Expect = 1e-19

Identities = 76/82 (92%), Gaps = 3/82 (3%)

Strand = Plus / Plus

Query: 2653 tctgccatgattgt-aagtttcctgaggcctacccagccatgtggaactgtgagtcaatt 2711

|||||||||||||| |||||||||||||||| |||||||||||| ||| ||||||||||

Sbjct: 663 tctgccatgattgttaagtttcctgaggcctccccagccatgtgaaac--tgagtcaatt 720

Query: 2712 aaacctctttcctttagaaatt 2733

|||||||||||||||| |||||

Sbjct: 721 aaacctctttcctttaaaaatt 742

Score = 56.0 bits (28), Expect = 7e-06

Identities = 28/28 (100%)

Strand = Plus / Plus

Query: 2650 ccttctgccatgattgtaagtttcctga 2677

||||||||||||||||||||||||||||

Sbjct: 623 ccttctgccatgattgtaagtttcctga 650

>5HSAR062721 BR396457 5'UTR in PREDICTED: Homo sapiens hypothetical gene supported

by AK093334; AL833330; BC020871; BC032492, transcript

variant 14 (LOC399753), mRNA.

Length = 2100

Score = 97.6 bits (49), Expect = 2e-18

Identities = 77/84 (91%), Gaps = 3/84 (3%)

Strand = Plus / Plus

Query: 2650 ccttctgccatgattgtaagtttcctgaggcctacccagccatgtggaactgtgagtcaa 2709

||||||||||||||||||| ||||||||||||| |||||||||||| ||| ||||||||

Sbjct: 628 ccttctgccatgattgtaa-tttcctgaggcctccccagccatgtgaaac--tgagtcaa 684

Query: 2710 ttaaacctctttcctttagaaatt 2733

|||| ||||||||||||| |||||

Sbjct: 685 ttaagcctctttcctttaaaaatt 708

>3HSAR062576 CR413216 3'UTR in PREDICTED: Homo sapiens hypothetical protein

LOC151234 (LOC151234), mRNA.

Length = 1403

Score = 95.6 bits (48), Expect = 8e-18

Identities = 69/76 (90%)

Strand = Plus / Plus

Query: 2657 ccatgattgtaagtttcctgaggcctacccagccatgtggaactgtgagtcaattaaacc 2716

|||||||||||| ||| ||||||||| |||||||||| |||||||||||||| |||||||

Sbjct: 577 ccatgattgtaaatttgctgaggcctccccagccatgcggaactgtgagtcagttaaacc 636

Query: 2717 tctttcctttagaaat 2732

||||| ||||| ||||

Sbjct: 637 tcttttctttataaat 652

>3HSAR052637 CR342155 3'UTR in PREDICTED: Homo sapiens hypothetical protein

LOC151234 (LOC151234), mRNA.

Length = 1403

Score = 95.6 bits (48), Expect = 8e-18

Identities = 69/76 (90%)

Strand = Plus / Plus

Query: 2657 ccatgattgtaagtttcctgaggcctacccagccatgtggaactgtgagtcaattaaacc 2716

|||||||||||| ||| ||||||||| |||||||||| |||||||||||||| |||||||

Sbjct: 577 ccatgattgtaaatttgctgaggcctccccagccatgcggaactgtgagtcagttaaacc 636

Query: 2717 tctttcctttagaaat 2732

||||| ||||| ||||

Sbjct: 637 tcttttctttataaat 652

>5HSAR057298 BR365291 5'UTR in PREDICTED: Homo sapiens hypothetical protein

LOC647570 (LOC647570), mRNA.

Length = 104

Score = 95.6 bits (48), Expect = 8e-18

Identities = 66/72 (91%)

Strand = Plus / Plus

Query: 2619 ccttgtgaagaagatgcttgcttccccttcaccttctgccatgattgtaagtttcctgag 2678

||||||||||||| ||| |||||||||||||||||| ||||||||||| || ||||||||

Sbjct: 33 ccttgtgaagaaggtgcctgcttccccttcaccttccgccatgattgtcagcttcctgag 92

Query: 2679 gcctacccagcc 2690

|||| |||||||

Sbjct: 93 gcctccccagcc 104

>5PTRR004722 BR196016 5'UTR in PREDICTED: Pan troglodytes similar to ADP-dependent

glucokinase; ATP-dependent glucokinase (LOC453727), mRNA.

Length = 531

Score = 93.7 bits (47), Expect = 3e-17

Identities = 56/59 (94%)

Strand = Plus / Plus

Query: 2635 cttgcttccccttcaccttctgccatgattgtaagtttcctgaggcctacccagccatg 2693

||||||||||||||||||||| |||||| ||||||||||||||||||| ||||||||||

Sbjct: 333 cttgcttccccttcaccttctaccatgaatgtaagtttcctgaggccttcccagccatg 391
