# Supplementary material for: The DMD Locus Harbours Multiple Long Non-Coding RNAs Which Orchestrate and Control Transcription of Muscle Dystrophin mRNA Isoforms
Source: PLoS One. 2012 Sep 21;7(9):e45328. doi: 10.1371/journal.pone.0045328 (PMC3448672; doi:10.1371/journal.pone.0045328)
Supplement: File S2 — Full FASTA sequences of the DMD lncRNAs completely characterized. (DOCX) [file pone.0045328.s013.docx]

File S2

>ncINT1Ms2

TTTTTTTTTTTTTTTTTTTTTTTTTTTTTTTAGTAGATCCAGTGCCCGGC

AAACAGTATGTGCTCAACATATGTTTATTTAATGCGACCAAAAGCATACA

ATATAAAGTGTCATTATCATTGCCACTGATATTTTTAAAAAGCCATGTTC

TATACTTATTTCATTTTTACGTAAGCCACAATTTTACAAGGTTTCAAACA

AAAAAAAAAACCCACACCTTGTTTTCTTTAATATAAAATATTTGGCAATT

ATAAGCATGGACCGTGCTGGCAGTAAGTTAAAATGTGACTGATTTTTTTT

TTCTCCGTTCCCTACTCTCAATTTTCAGATGCGGAAATTTCATTTGGAGA

GAACAGGAGGTGTTGGTGGGAAAAATCTCATTAATCGTACCGTATTCCCT

TGTCTACAAAGAGTACTGCATCCAAATTACAGAAGAACTAAAGAAACCTT

GTAGCATGAGTATAATTAGCAGTGCTACTAAGTGAATGGAAATGGAGGTT

GCCTAACTTAATTAACTGTAATCATTACTCATTGTCGTACTGCTTAGAAA

GATTAGTTGAGGGTATTTCTTCTGTGGTATTACTTTAGGCGTTTCTCCTT

CCCTCTTAAATGTGAAACAATTACACTATAAAGAAAGAATTAGCATGCCC

TTTCAATCAGATAGAAGCTTTTCCGAACAAGTAAGTTCCATGTCCTAGCT

CGAGGGCGGCAAAACAGGAATGATGGGAAAATATTTAGTTGGGCCAAAGT

TAGAGAAATTAAGAAAAAAGTAGTTAATTGCAGAAGTTACTGACTTGAGG

AGGAAAAAAGGAACCCTATATATGAGTTTCTGTAAAGAAATTAGCTCTTG

GCCAGGGAAGAAATAAATAACAAATTAAGATCTAAAGGGAAAAATTAAGG

ATAACACAGTACAAATACGCAGTACTGTCTGATGAAGTTGTTCACAATTG

CACAACAGTGCAAGATTCCACAATGCACCTTCAAGGACACTGTAGCTAAG

GATTGCTTTCTGATCATTTATCCTTTTAGCGGAAGACACTTGGCATCTGG

GAACATTTGTTCTTTTGAATAAAAATCACCTCATACCTAAAAGGAACCTT

TATAAATATCAGAAAAACAGGAATGTCTGTGACGACGATCATTGGTGGGG

CTTGGCCTCATTAGCTCTGGGGCCACTAGTGTAAAAAAAGGTTCACAGGG

ACTCTCTTCTAGTAGGGACTATATATATATATAAATATATATATATATGA

CGGAGTTTTGCTCTTGTTGCCCAGGTTGGAGTGCAATGGCGTGATCTCGG

CTCACCGCAACCTCTGCCTCCCGGGTTCAAGCGATTCTCCTGCCTCAGCC

TCCGGAGCAGCTGGGACTACCATGCGCCACCACGCCCAGCTAATTTTGTA

TTTTTAGTAGAGACGGGGTTTCTCCATGTTGGTCAGGCTGGTCTCGGACT

CCCGACCTCAGGTGATCCGCCTGCCTCGGCCTCCCAAAGTGCTGGGATTA

CAGGCGTGAGCCACAGCGCCTGGCCGGGACTTCATATTTAATGAGGCTTT

TCACTGTTCTAGGAATTAAACTGAGTTTCAGGTATTGCATTCAAGAATCT

TCTCAGCAAACCTGCAATATTGGTAATGTTATTCCTTAAACTCGTTTTAC

AGGTGAGGAGATTGGAGCTCAGAGAGGTTTTTCAGTCATCCAACTAACCG

GAAGTGCTTAAGAATCCAGCTCTACCTGACTCCCTAAACAAGTGTGTGTC

AAGAGTTAATGGGCATACAAACCACCTGGGTATCTTGGTAAAATACGGGT

TCTGATTCGGTAATTTTAGGTGGGACCTGAGATTCTGCATTTCCAATAAG

TTCCCTGGGGCTGCTGCGGCTGCTGCTGCTGCTGGTGGTCTTTGGACAAC

ACATCGGGTAGCGAGGCCCCTAGACCCGTGGTTCTCCAACTTTGCTGATA

TTAGAATCCCCTGAAGAACTTCTTTAAGAAAAAAACCCAGTGCCCAAACT

GCACTTCATATCAATGAAATCAGAGTCTCTAGAAATGAGACTCAAGCAAT

TCTAATGAACAGCCAAGTTTGAGAATCTGTACCCATTGACATGTTGTTAT

CAAATTAGGTGCTCATCAGAATCAACTGACGAGCTCATTATAAACCCAGA

GACCTACATTCATTAAAGCAGTGATAAGACTTAGGCCTTTGCACATTTAG

GTAGACCCCCCAGGTGATTCTGTTATCCAACAGTTTGAGAACCACTTTCT

AGACCATGCTCCTTCAAATCAATCAGACCAAGCCCTGCAGACAAACTTAC

CATTCAGGCAAAAAAAGTTGTGAGTAGGAGCTTGTTTTCATGCATATCCT

CAGGGTTCATCGGTACGATAAGATATCCT

>ncINT44s

TTTTTTTTTTTTTTTTTTTTTTCAGGCAATTCAGTTTTCAGGAAGAGTAC

AAAATAAAGTAGAAGATCTAGAAATAAATTCCCTTAAAACTATCTGTGCC

TACTCCTTGGAATGCCTTCATGTACTCCCAGGTTACTCCACTGTGAGGAC

AGCTGTTTTAAGAAATTTCGAATCATTACCTGTGACAAGAAATATAAACT

GGCACATCAGAATCTCCTGATAACTTGTGTGTCCACTTCCTCAGTTGTAC

CTCACAGCTAAATAGTTATCTGTTAAACCAAGAGAAGTTGCAGTTCTGGG

GGCAATAGCCTTATTGGTATTTGTGCCTGAATATAATTCTGAACTCCCTC

TTTTCTACCTTCTAGGCTTTGAGTTCATTTATTTCAGCAGCAAAAGATGT

CAGGAGCAAGAGGCAGTCCCACATAGTGTAGATTCATTGAATGAGGGAGC

CAAACAGTGATTTAGAAACAACATTGCCAGTAACTATCTGATTAGCCTTA

GGCAAGTTTCTCAACCTTCCTGAATCTGTTTCTTCACAATAACTCCCAGT

CTGGCTGCACCTTCGACTCACACAAAGATCTTTAAAGAGATACTGTTGTC

CAAGCATCGCCCCCAGGAATATGGATTTGATTCTCTGCTTAATTCTAAAG

TGAAGGCAAAACAGGAAAAGTCAAAGTTAGAATCATTATCCATATAGTCT

TTCAAAAAATATATAATAAGTGCCTATCATGAGCAGTGTTGCAAGTTTTC

TGTGTTAAAATATCTACAAATTATACTTCTAAAGAAAAATTGAATGCTAC

TTGAATACAATAAATGATACATTTGAGCTTATCAGTCTTGAAAATCCTTT

GTTCAAAAACGATGCAAAGTAGAGAGGGCAATTTAGTCTACAAAAACTTT

TATTTAAAAAAACTGAATAAATAGCACAGACACATCCAAAAAGTGTATAG

CATGGAAAAATTCAATAATGTATAGCACAGAGACACCCCCAAATTTGTGT

TGTTTATCATGTTGCATCATGGTCTTAATTCCACATTTCATGTAGCAGAA

TGTATCCCTTCATTTTGAGATCCAGAAGGACAAAAGGGTTGCACAGTTCC

CCTTTTTCTGCCTCTCGGAGATCTTAAAAATGAATGATAAACAATTAATT

CTACCCAGGATACCAATGAAAATCTATTTTCTTTTCTTTTTCCCAAACAA

AAACCTCAGAAGGCAAGTAAACTGACCTTTTAAATGAACAAGATCTGACA

TGATTGAGCTAACCCAAGAAAGAAAATTCCCCTCTGAAGTGAATAATTTT

TTAATTAAAAGGTGAAATCTGACACATCACACTGGTTTTGTTATTATTGT

TGTTTTCTTCCATTAATCTCACACTCGTAAGAAAAAAAATGAGTGTATTA

TATATGACTGTGTTTTATAGATGCATATATTCTACTCTATCCATTTCAGT

CCATCTTTTCTTAATGCATTTGTCCTGTCACTTTTACTCACTGTAATTCA

ATTTTGTTAGGAAATGTAACTCGCTGTTTTATGCTTTATATATTTCACCT

GATGCAAAATGGACTACCTGATTGATTCTGCATTTTTAGTCCACCTTTGA

ATTTATAGATAAAGATCAACAATTAACTTTAAGCATTATGCTAAAAAAAT

CTTAGCCAGAAGGATTGCTTCCTGAAAATGATTTAATTTGTATTTTTCTT

AAATATAATAAAATGCCCTGATAAGAAGTAATCAACAATTTTGATTCTAG

TTTTCCATTTGATTTGAACATGTACATTATTTCAGCATGGTGGATGATAC

ATATCTACAGCTTGAAATATGTGCATTGGGGAGGTTTTGTTGAATGCTAG

ATTGCTGTTTTGCTGTAGTTTGATTTATTTGTGTGAGTTATTAGCTTGAT

ATTGGAAAGTAACTAAAATTTGAATATATGATATCTCTGCTTCTGAAATA

AATATAGCGATTGAAAATGGACCTTTTATGAACGTTGAGTGGGGCAATTT

CTGGCAGGTGGGGAAGAGAGTAAAGAAGATTCATTGTATGAAAGCAAAGC

AAATACACATAGAACTTGTAATTTAACGGAACATATCAGCAGATGTGGTT

CTCAGAATGACTCTTACATTTACATTAATGACTATCTAAAAAGAATTAGA

CAGCACCGTGAACTTCCTTTTTCAAAATTAAAAGTTTTTTTTAAAATAAA

GAAGTTCAGATTTTAGCATCAACTGACCAAAGCATAAGGGGCCATCCAAG

AAAAAAAATAATCTGAAGCACTTTCATAAAACGATGTTACAAATCAATAT

TTTAGACACTTTGAAGATCATCTCCTTGCTTTTCAACCTTCACACTTCCG

AGACAGGGGATATATATGTTTATATCATCTCTTTTTCTAAAATAAATCAT

TTCTGATTGAGCATCTTTTACAGAAGAGCCCACTGCAAATGTCAATTTTT

ATAATTGCCCAGACCATACTTGTGGGAAAAATGAAGGGACAAGAGTAGGA

AGGTCATTCAGGGTCACTGAATACATTTCTACCACAGGAAAGCATTACGT

CTTTTGCACTTTATTTCTTCAAGCCATTCCCTTCCAACATCTCATATCCC

>ncINT44s2

TTTTTTTTTTTGGCAGTTCCATTCTCCACAGGTGATCTATAGCTTGAACT

AAACCTAAAGCCATCAGATTTTCCAGGTTTAAAACATTTTAAAATGGTCC

CATCTAATCCACAGGTTTTTAAAGTTACAGTTAATAATCATGTTACTGAA

AACATACACTAGAAGAAATGTTATCAAGACATTAATTAAAAGTCATGTTG

TTAAAATATATACAAGACATACACTAGAATTTTTAAAAATATTATTCACA

TACTAATAAAATGAAAGTAAGGAAATAATACAATAATTACAAGAACTTCA

AGAATTTCTCATAGCTAGTAGTGAACATACTGATCAGTATAAGGGCTTGG

GGGTTTATTAATATTTCTGAGGCATAACTGTCTTAAAAAGAGCCAAAAGT

GAGATCTGATAATTTATCAGATTGTCTAAGAAAATGTTAATATTCATCAT

CTATAAAATGACTAACTTTTCTTTGGTGTACATGGTTTTCAAGAAAACAT

GATTCATTATTTCAACATTTAAAATTTGCCTAGAGAGCAGCAGCATCCCT

GACAGACTGGCTCATGCTATGATTCGGTGTCAGGATGAAAAGGCTGGCTT

AGAGACATGTTCTGGGTTCAATTTCCAGTGGTTTAAAAGATTTCTTGGAG

TTAGGTTGTGCATGATAATGTGCCTCAATCTAGAAGAGGCATGGGCAACT

CCCAGCATGCAAGCAGAGCTCCTGGTTAACATCTGAAAGTATTCACCCAC

ACCGTAATCCGCTGGTTTATTCCACCTTTATGAAACTCCTATTACATTTG

TCCACCCTGTGCTTAAAGCAGCAACAGCTTAAAAGAGGGACACTTCAGAC

AAGGCCCCTTCTGGGTCTCTTTCTCTTTCAGTACCGCAGAGAAAAAGATA

CAATGGAAGCAACAGCAGTAGATGGAATTTTTATTTGAAACACCACCACT

AAATAGCTTTCAGATGAGGAATACAATTCTTTGGTATTTCTATTTTAAAT

AGTGCAGGGGCTGGGCTATGAGATTTTCATATGCTATCTCATTTATTTCA

TTTATCTATACATGTTTAATTTAGAGTGATGATGTAACATTGGATGTGGA

GACTCCAAAGCTGAATAATGTAATATATCAATACAATACCTGCAGAAACA

GCTATGCAGTGGAGTATTTTGGTTTTATACTTCCTCAAAAATGTTCATCA

CGTTATTTTCACTTTTTTTTTTTTTTTTTTTTTTTTTTTTTTTTTATAGA

AACACAGGACTGGCTTTGGAGTTATTTAGGGGAAGAGTGCCTTTGGTCTT

TAGAGAAATCTTCCTGGACAATTCTTGTAACAAATAAAACATCAAGGTGT

AATTATATAAAATCACTATTAAAACGCAACTTATTTGTACTATATTTTAG

CTGTTTTAACACTAAGTGACCTTACACTCATTCATTGTTTCTATTGCTCC

CTGTTATTGTTCCTTTCTAAAAGAATGTTCTCCCTTATATACTGTACTAT

CACCACTACTTAATTAGTTTTTGTATTCAATAGTCTTGCTCATTGGCGCT

AACATTAAAGTATGAAATAAGTTAAATATAATGGAAAAAATAATTTCTTA

CTTTAATAGTCACTTTAGTCTTCACAATAGAATTCTTTAAATCTATAACT

CAATACTTAACAGCTGAGAAGAATATGAGTGTGTTTAATATAAATTAAAG

CAGCAATTTCTGTTTCATACCACATTTTCTTTTCAATTCATCCACTGATG

GACGTTTAGGTTGATTCCATATCATGGCTAGTATGAAAAGTGCTGCAGTA

AACATGGGAACAAAGCTATCTCTTCAAAGATACTGATTTCCTTTTCTTTG

GATAAATGCCCAGTAGTGGGATTGCTGGATCATCTGGTAGCAATTCATGT

TTTATTAAGAAAGTGTTTACAACACAGAAGCTCAAAAGCCTTCAGCTATC

TCTATACAATGCCATTTTAAACACCTTTGGAAGATTATTAAAATATGTTA

ATTATTTTGTCGAAAAACTAAAAATTTAAAAAGCAAGACAGGTCAACAAA

GGCCAGATTAGAAATTACCATAAATTATTAAAGTCATAAAACATACTGAA

ATCTTTGGGATGTAATTTAACACATGAGATTTACGACTACTCAAGAAGCA

TACTATGTAGTATGTCACTTTTGTGCTTTGAGCAAGGAACATACTTTGAT

TCCTCTATTTCAGTGGGATGAAAACAGCTTGTGCCTCTAGAATTAAGACA

ATGGCCCACAGAATTAATAATATGATAATGAATTAAATTCTCTATATTTC

ACAGAAAATAACAAATTTTTTATGACTGTCAATAAAACCCTCAGATAGTT

CAACTCTGTATAACTAACAAGAAAAATTAGTTTAAAATGTCATTTTAACC

CTCCTTTAAAAAAAAAAGCTTATCTAAATTTACTGGAGATATCAATGAGG

TTACGGAGAATTATTTTAAGAAATGTAAAAAGGAACCACAATGGTTCTTT

GCTCATTAACAAAATGCTACATAACTACAAGAGATGGTTTATAATTACAA

GAGGTTAATCATGCTTTGCAAGTTGTAAAATTTACTCTCTTAGGAAATAA

TTTCAAGAGTCCTACTGATTGTTAACAGGTTAAAAAACCTTCAACGTTTG

TTAACACATTAAAAAGTCTTCACATATTTGGCCTTTACCAAAGTAGGCAG

TAGTGACCATGGTCACCT

>ncINt55as

aatttctaaaggaaagaggtttaattgactcacagttccacatggctggg

taggcctcaggaaacttacaatcatggcagaaggtgaaggggaagcaagc

atcttcttcacaaggccgcaggaaggagaagcgcccagcgaagtaggaag

agccccttataaaaccatcagatcccgctatcatgagaacagcatgggag

aaactgcccttatgattccattacctccacctggtctctcccttgacacg

tggggattatggaggttatggggattacaatttaagatgagattgtgggg

tggggacacagccaagccataccaaaaactctgttttttgtttttgttta

atggaaatgatttagaactttattttctgatgtttctttttcataaaacc

acgacaccaaaatctacttttcactgctccattcaactagtagagaatat

ctaatctcttctcaagtatttctttctcaattatggtggttttagctaag

aacagcttatggcatgcttttctaaataatattagaacacataaattatc

tgtacctggtattaccacattcattgctcattttaagatctcaattgata

cattcaattcatatatatttaaaattgattcatttagagcaagagataca

ggcattttaatgtattacactgctactaaagcttagcaaattattctttt

ttgtgcccacaaattatcatccattcatgtcctaaaaataaaattgaatt

tattatactttcccatttatccaaaaaaaaggttttttttaacaattgat

gcagatacacattttcaagctaaaaatatgtgtgaaagtggcctctttct

catagtatttattttaggagtctagcaataatttttcttaggttatcagc

acatgtcttagcctgaattatttgaattcagtctgtgtcttcaagttcag

atggttatgtgatcttgttaagatctcaaagtagtgggaatgatggagta

tacaacaacctcattgttttttatggcaactgtcatttactgaaggacat

aaggctagcagaacatggtcagagaaggaatcaaagtttggtcagccaac

tctgctccacagctacaagctgctagacaggcataaatttttccaaacct

acacaaagggacttagggcccttggctgagagcgacattctaaccacttc

cttatttatggctggtggggtttgtacattttctcatttctgtataacat

ttcttgactgtaataagcaatgtattcattctgctttaccactttcacta

accttaacctcaatatatactcaattaagcaattgaaaacagcagtttta

atcttttgacataaatgatttcctccgaagcaaaatgctggaaatcccct

caaatgcaccttttattgatgaatacctataagcaccacctacagtcgct

ggaggctgacaggaaccaaacttgatgataaccactgagctgagaatttt

caactcactctttttccctgtatggttcttctagctgcattatttcccac

tatttaaagctacagctggtgaactattcaaatatttaaactttggagaa

gaaaatatcaacttatcacaaccctctttttatattctaaattcatatac

ctgtttggtacttaaaggaaaaatatgctgaggaacaggctggtcataag

actgtatagaacgtgcatcttccatcctattgaggtgactcctagacaat

gggaaaaatgccttcactcgacttgctcattaaatgtgaccgtagctgct

aatcttttggcgctgtctcgaactttaattagatgtgctcttctcttgaa

ggttggaactacagtatccagagaccatagaatcacagagttgaaaacaa

aatcttggaaatcattgaatccacttatcagatgagaaaaaaaaaataag

cccatggagatagccattttaaaacatatcattctatttagcctccaatg

taaaacaatgagttactatgtttcaataatgttgatgttaagaaattatt

tgatagcttcctcacttggtctcctatattcctccaaggttactagttag

gaagactgtcattcaaatttggagactacataagaagcagaaaaagcata

taaagaggcacatgaaattggaacttttctggtaaaatcttctttcttaa

actctcctcaaataagctgttggtggcaggaggtgaaagacagcctccac

cctttagcacagtccgtacttgtcagcatttcccaggaagggtgatgtct

ggaaatgatagagattgtggaagcacattgcattatgggtcaagaatgcg

aaggtcaaggagtggagtcttcctttacgaagtagtgttaactgcttggc

gtggcattgttgtaaacagaagccaccaggaaggatcatccttaggaggg

aacctgtagatatgacttttcaacaaatggtcctgcagaaattggacatc

ctttcatgcttcacctaagtctttttttcttttctccatcactcagttaa

gagcttagttctggaggatagaagtctgagatcaaggtgtcaggagggtt

ggttccttctgagggctcggaaggagaatctgttccattccattccccta

gtttctgatggtttgttggcaatctttggtgctccttgtcctgtagatgt

ctgccttcattttcacatggcatgccccctgtg

>ncINT55s

GTTTTTTTTTTTTTTCTTTTTTTCTGGGCTCTGAGAAACCTCTCTCCCCA

ACAACGGCTTCAGGTCTAGGAGAGGTGACAGCTCTGCTGCTGCTAGTCCT

AGAGTCTTGTGCTATCTTGTGAGATTTCCCTACACCCTGCCCAATCCTGA

GTATGTAGTGCCTTGTAAATAAAGACTCCTCAACTTACCCTAATCTGAAT

GTGCCATCTGTTTCTTGTTGGGACTCTGACTGACTGAGATATCAACACAA

ATACAATCATGAAACAGGACACAAATTCAGCACTTCACAGAGTTATTGCG

GTACTATGTTAATGGGCATGAAAGTTATCAGTAAACTGTAAGTGCCATAC

CGATGTTGATTGCTATCATTTAGCACAAACTATGAAAAGTTCAGACTATG

TCCATACACAGGTGTCTCAACACAAAACCAGTTGACATGGTCTAACTTGA

TATAGGATTGATGTGGCTGTACTGTAACAGTTGTCCAATAATCATGTTGT

AAATAAGTTTTTAATATATTAACAGCATACTATATTGATACATATGTAAT

CACAGTGTTATCAGACTTATCCAGTGTTAATATGGCTGACCATAGACAAG

GTCTCAATATTCCATTATCCGCCAGTCTGTTCCAGATCGTGGGCCACTTG

TACATGTTCCTAGTTCGGAATCAATTAAGAGACTAGCTCTGTGAAGAGAC

GGGAAGCATAACCTTTTTCTGAGTTATAAGTATAAAGTCAGCCCTGCATT

CTTTTACCCTGATTGGGGCTGAGCTACATTACAAATCTCCTTTGTCTCTT

CAAGCTTAGGGGAGCTTGCAGGAAATTTTCTGTTAAGAATTTATGTTTTT

ATGAAAAACAATGATACTGAGACACAGAGTGTATGCTACGAAAAAAATAA

ATACTTTTAGGGTAAAAAAGGAAAAAGGTGGTATTAAAATAAATTTTCCC

AAATACTGCTCTTTATATATTTCGTTAGTGACATCTACACAATAACCATA

TTTTGGGAAGCAATTAAACACCTCACATGGACAAAAATCATATCGGTTTC

TTACTATTTGGGCTGGCAGTACCTTATAAAAAGAAAAAAGCATCAAGGCA

CTGTAGTATGATATTTTTGAGGATGTTTTTCCTATAAAAATCTTCAAATG

AGCTGCAAATGGACCCAAACAGACTTATTTTAATATACTCTAGGACCATA

TATAATAAAAGGTCCTATACTATTTTATAGAAATGGCTTCTGTTGCCTTA

GATGAGGTTTACACATAAATCTTTTAACTTTTAGTGCTGCTTGTTTCACT

TCTGGTGGATTCTTTTTACAACAATGCCCTGCCAAGCAGTTAATACTACT

TCATAAAGGAAGACGCCACCCCTTGACCTTCACGTTTGTGACCCATAATG

CAAGGTGCTTCCATCATCTCTATCTTTTCTAGATATCACCCTTCCTTGGA

AGTGCAGACAAGTGTTTTGTTTATTTGTTCTTGTTTTGTTTGAGGTCAAT

TCACTGGCTCCTTTCTCCAGTTTTTTTTTTTTTTTTTAAACACCTTGAAA

GCTTCTAATACATCTGCTAGCCAAAACAGTCATTTTCATTACATGCTTGT

GGCTGGTCAAAAAGAACAGTTGTATAATTTTTATTTTCTTACCCAGACCA

CAAACAGGTTATAAAAAGTACATGGCCCTTAAATTTTGGCGGTTCCTTTA

AAATTTAGTTCAAAAGCCAACATGAAGAGTCAGATGGAATGACTGTCACA

TGGAAGAGCTACCAAGCAGATAATAAGTTTAGAACTCTTAATTGAAAATT

AACACACCTACATTGTATTCCCAAATTGAAGAAAACGGCTGTTTTGGCTA

CTGCTATAATAAGATGCTGCATACTTCACATGCTGAATCTGGAAGGGGGA

GCAATTCAGTCCTATCTGACGGTTGTTCCTAAGCTTTAGTATAACTTCAA

AGATGAGGGTATGAAATTCCGTCATCGTCAGAGAGATCCTGCTTTCTCTT

CCTTGGGTTGCAAAGTTAGTAAATATTAGCCTTCCATTTTAACTAACTCG

TTGAGCTTGTCTCCAATAGAAGATTGAATTATTGGCCTCAATCCTTTCAC

ATCCCTAGTAGTATCATACATTCACGCTCCAGCCATGGCCTCACATAGGT

GGAAAGTATTTTCCTACCCCTTGACTTTGGGCTTGGCCACAAGACATGTT

TTGTCTAATGGGCTATAAACAAATGTGACAAGAGGAGAGATTTTGAAAAG

TGTTGTTTGTGCTTCTGTCATTGCCTTGTGCTACTCTGTCATACCCCGAG

TAGCTGATGTCCCTTCAGCTTGAGTCTCAGAAGAAAATATGTGGTATAGA

CCCGAACAGAACCGACGGCCTAGAGATAAGCATAGCTAAACCATTTCCAG

CCATGATTGGCCAAACCCTACCAAACTGC

>nc3UTRas

TGGTGGTTATAAAGAACACAACACGAAATAATGTCCAAATTAATTATGCT

TAAAATGCAGCAATAAAGCTCTCAATTTTTGTTCAAATATTATGACAGAC

TCACTCCAGAGCTAATGTGTCTAAAAGAAAAACAAAAAGATTAAAACAAA

ATTATTTATGCACTCTATTTACCTCTGATTTTAGAATGAAACTTACTTAC

TTAAACTTCTTAGTAGGATGTAAAGTAACCCCTTGTTTTAAATCTGAGTT

TTAAAAATCCTTGGGTAAAGAAAAGGTCCAGCGTCACATAAAGGAAAAAA

ATGCAAGACAAAAACCAAATCTTCATGTAATTTGGTAATTTGTTACCTTA

GAGCTTTGGGTTTTCTTTTGAAAATTATGAAGGAAAAAGAAAGAATTATA

AAGGAAAAAGAAAATAACGCAATGGACAAGTGGTGAAGCTGTGAACTCAG

GTGTGCACAATTATCAGGAACACCCCAAAACCAAAGTGAGGTAGAAATAG

CATGAGAAGCCGTGTTTGATGTTAATTAATTAATTATTAATAATGGACAA

AACCCACTCTCCAAAAGCTAATTACACTTGATGTCAGCCCACTCTCCAAA

AGCTAATTACACTTGATGTCAGAGGTAACAGATTTGCAAAATTATAGGTC

ACACGGTGGTATCTATTGAATGAATGATTTAAAAATCAAAAAGAAATAAA

ATGGCATGAAAGAGTAAAGCTTTTTCCTACCAGTCCTTAGCTTTTCCTCT

TGAGCTTTTCTCCTCTTTTTTGAGCAATTTTTGTTTGTTTGTTTATATAT

TTAACCTGTCTAATCCACCAAGAAGGGTTTTTTTGTAACATTTGAATCAA

TTTGCCTTCTTTCTTACTTACTTACAAACCTGTGTGGAACTACTCGCAGA

GAAATGCAAAGGATGGAAACACAGTTCATGGGCTTCTGGGTTGATACCTG

TCAGTATCACAAATGTGATGGGGCTACTGTTTTACACCTTTTCCCAAAGT

TTATTTATTTTAAATTATGTCTTGTGGCATTTAAAAACTCATCCCACATG

GGACAATAAATCTAAAAAAGCAACAAAAACCAAACCACCCAGTTCCTTTT

GACTGTGAGAAGAGGGCATAATAATTTAGTTGTAATTACAGAGAACTTTA

TGTATTATGAACAATCATCCAATCCTTCACTTAAAGAGTGGCCTACTCCT

TCACAGGGATGGGCTGGGAATCCATAGCCCTCCAGCCCTATCATCGTCCT

GCTACTGCTTGGGAGTTAAAAAATACCTTCTGATGTTCACAAGGTCAGAA

TACCTTCTGATTGATTTCCACTGAAGCATCATTAAATCTAAATCGTGGCA

TTGCTAGCAGCAGGAAGCTGAATGTATCAATCAATCAATCAATCAACCAA

CCAACCGATTACTCACTCTGATATAATAAGTCCTGTGTATTCATTCACAT

GTTCCCTTTAAAAAAATCCAATACTTTACTTTACTTTCGTTGTCAGTGGA

AAGTTGTTTAAAATGAGAAACATCTGGAGCTGTAGACAATGTTTTAGTGT

GGTGTAGTTTCCTCCTGGCTTCCGGCTCCGGGAAAAATCCATTCTAGACC

AAGCAGGTAAGCCTGGATGACTGACTAGAAGTAATTTCTTTCTATTAGGA

TGTGACATGAACATTTAAAAAATGGAAAAAGTCAGTCTATAGAAATTCGT

ATCTCTTTATCTATATAACTATAGTATTTATATACTTATAGACATATAGA

TATATGAAATGAAAACTCAAGCCTGCCCCACTCAGCTGACAGTTCTCAAA

TGAGCAGTGTGTAGTAGTCATTTGGTGTGGTGGTAGAGGAAGTCTTATCT

TTAATATGCAAAAAAAGAAAAA
